# Impact of posttranslational modifications on atomistic structure of fibrinogen

**DOI:** 10.1371/journal.pone.0227543

**Published:** 2020-01-29

**Authors:** Žofie Sovová, Jana Štikarová, Jiřina Kaufmanová, Pavel Májek, Jiří Suttnar, Pavel Šácha, Martin Malý, Jan E. Dyr

**Affiliations:** 1 Department of Biochemistry, Institute of Hematology and Blood Transfusion, Prague, Czech Republic; 2 Department of Biochemistry and Microbiology, University of Chemistry and Technology, Prague, Czech Republic; 3 Proteases of Human Pathogens, Institute of Organic Chemistry and Biochemistry ASCR, v.v.i., Prague, Czech Republic; 4 Military University Hospital, Charles University in Prague, Prague, Czech Republic; University of Pisa, ITALY

## Abstract

Oxidative stress in humans is related to various pathophysiological processes, which can manifest in numerous diseases including cancer, cardiovascular diseases, and Alzheimer’s disease. On the atomistic level, oxidative stress causes posttranslational modifications, thus inducing structural and functional changes into the proteins structure. This study focuses on fibrinogen, a blood plasma protein that is frequently targeted by reagents causing posttranslational modifications in proteins. Fibrinogen was *in vitro* modified by three reagents, namely sodium hypochlorite, malondialdehyde, and 3-morpholinosydnonimine that mimic the oxidative stress in diseases. Newly induced posttranslational modifications were detected via mass spectrometry. Electron microscopy was used to visualize changes in the fibrin networks, which highlight the extent of disturbances in fibrinogen behavior after exposure to reagents. We used molecular dynamics simulations to observe the impact of selected posttranslational modifications on the fibrinogen structure at the atomistic level. In total, 154 posttranslational modifications were identified, 84 of them were in fibrinogen treated with hypochlorite, 51 resulted from a reaction of fibrinogen with malondialdehyde, and 19 were caused by 3-morpholinosydnonimine. Our data reveal that the stronger reagents induce more posttranslational modifications in the fibrinogen structure than the weaker ones, and they extensively alter the architecture of the fibrin network. Molecular dynamics simulations revealed that the effect of posttranslational modifications on fibrinogen secondary structure varies from negligible alternations to serious disruptions. Among the serious disruptions is the oxidation of γR375 resulting in the release of Ca^2+^ ion that is necessary for appropriate fibrin fiber formation. Folding of amino acids γE72–γN77 into a short α-helix is a result of oxidation of γP76 to glutamic acid. The study describes behaviour of fibrinogen coiled-coil connecter in the vicinity of plasmin and hementin cleavage sites.

## Introduction

Posttranslational modifications (PTMs) may lead to alterations in the protein secondary structure as well as their functional and binding sites by changing the charge and/or structure of their amino acid side-chains [1]. At low concentrations, PTMs naturally occur in the human body where they participate in various physiological functions such as cell differentiation and gene regulation. At high concentrations, however, they may indicate serious diseases such as myocardial infarction, venous thromboembolism, arterial and venous thrombosis, pulmonary embolism, and cancer [2–8].

PTMs can be introduced in the protein structure either enzymatically or by reactions of amino acid side chains with free radicals and other reactive species. Nonenzymatic PTMs are usually a result of protein interactions with various reactive oxygen, nitrogen, sulfur, carbonyl, selenium, chlorine, or bromine species under physiological conditions [9]. Higher concentrations of these elements may lead to an imbalance in oxidants and antioxidants in favor of the former, causing oxidative stress. Among the blood plasma proteins, fibrinogen is known be the most frequent target of PTM [10].

Fibrinogen, one of the most abundant blood plasma proteins [10], is the final member of the blood coagulation cascade. After enzymatic removal of fibrinopeptides A and B [11,12], fibrinogen is converted to fibrin that polymerizes into complex fibrin net [13]. Fibrinopeptides are cleaved by thrombin under physiological conditions and can be removed by other serine proteases of snake venoms as well [14]. Fibrin net is, together with platelets, erythrocytes and a few leukocytes, the major component of thrombus [15,16]. Under physiological conditions, thrombus prevents blood loss at the sites of injuries, but under pathophysiological conditions it can stuck blood vessels (thrombosis) and its release into blood (embolism) may result into stroke, myocardial infarction etc. [17,18]. Fibrin net is enzymatically lysed (fibrinolysis) mainly by plasmin [19] and fibrinolytic enzymes are contained in saliva of some parasites. Hementin is, for instance, a fibrinolytic enzyme of Amazon leech *Haementeria ghilianii* [20].

Fibrinogen is composed of a pair of heterotrimers, each of which is contains Aα, Bβ and γ chains [13,21,22]. Mature human Aα chain consists of 610 amino acids and can be divided into fibrinopeptide A (16 N-terminal amino acids of the Aα chain), that is cleaved out during conversion of fibrinogen to fibrin, and an α fibrin chain, that remains in the fibrin hexamer; for fibrinogen notion see Medved and Wiesel [23]. The most prominent structural feature of the Aα chain is an α-helix (amino acids AαG48–AαR159). The rest of the molecule, but for a β-hairpin (AαK444–AαV464), is disordered [21,24,25]. Aα chain is in approx. 1–2% expressed in a form having 847 amino acids, that contains fibrinogen-related domain (FReD; amino acids AαR611–AαL844) on the C-terminus of the major splicing variant. This extension is known as an αE region [26]. The most prominent secondary structure feature of fibrinogen FReD is a central 7-stranded β-sheet that together with two short α-helices and a β-hairpin forms a B-subdomain. A-subdomain, that contains N-terminal part of FReD, is composed of three β-strands. P-subdomain, that is inserted between 6^th^ and 7^th^ β-strand of the B-subdomain, is mainly disordered, but for two short α-helices and a β-sheet [27,28]. P-subdomain contains Ca^2+^ binding site and a polymerization site. The only expressed splicing variant of the Bβ chain has in its mature form 461 amino acids. Similarly to the Aα chain, Bβ chain comprises fibrinopeptide B (14 N-terminal amino acids), that is cleaved out during conversion of fibrinogen to fibrin, and the adjacent fibrin β chain. From the structural point of view, the disordered N-terminal of the Bβ chain is followed by an α-helix (amino acids BβG79–BβC193) and a C-terminal FReD (amino acids BβS207–BβP456). Fibrinogen chain γ, that does not contain any fibrinopeptide, has 411 amino acids and is in approx. 8–15% expressed as a γ’ form having 427 amino acids [29,30]. A homologue of the Bβ chain [31], its major structural features are the N-terminal (amino acids γC23–γE132) α-helix, that is interrupted by a disordered loop between amino acids γY68–γM78, and C-terminal FReD (amino acids γT149–γG388). The others part of both γ and γ’ chains are disordered.

The N-termini of Aα, Bβ and γ chains are oriented towards each other. The C-terminal FReD domains of the Bβ and γ chains are at the edges of fibrinogen, while the C-terminus of the Aα chain loops back towards the N-terminus. N-terminal and C-terminal regions of fibrinogen are connected by a triple helical coiled-coil connector, that is at its N- and C-termini stabilized by disulfide bridges [21]. After assembly, fibrinogen molecule is glycosylated at positions BβN364, γN52 and eventually at AαN667 (i.e. in αE region) [32].

The impact of oxidative stress on fibrinogen has been widely studied *in vivo*, *in vitro*, and eventually *in silico*. It has been found that oxidative and nitrite stress increase the carbonyl count in a dose-dependent manner [33,34], alter the architecture of fibrin clot [35,36], and change the fibrin polymerization rate [34,37]. Moreover, modifications in fibrinogen increase platelet aggregation and decrease the efficiency of the tissue-type plasminogen activator that converts plasminogen to plasmin [38]. The extent of these changes depends on the nature of reagents and the length of fibrinogen exposure to reagents [33,39]. Reports have revealed that fibrinogen oxidation [35], nitration [4], and its reaction with reactive carbonyl compounds [40] do not change its secondary structure within the detection limit of circular dichroism.

To simplify the complex (patho)physiological states in the human body, various reagents known to be linked with certain (patho)physiological processes are used *in vitro* to describe the effect of the given reagent on the molecule of interest in a less complex system.

Hypochlorite (ClO^−^) is generated in an intermediate form of hypochlorous acid (HOCl) at sites of inflammation, injury, or infection as a part of the natural immune response [41]. It was demonstrated [33,35] that hypochlorite reduces the lateral aggregation of fibrin resulting in thinner fibrin fibers and fibrin clots with altered pore size [33,35]. It increases the clot lysis time [35] and stimulates dynamic adhesion of platelets [33]. Furthermore, it was reported that methionines AαM476, BβM367, and γM78 are more prone to oxidation by HOCl than methionines BβM305, BβM190, and AαM91 [35]. Recently, Yurina et al. [42] revealed that hypochlorite concentration of 500 μmol/mg fibrinogen, but not 50 μmol/mg fibrinogen, leads to fibrinogen fragmentation. Certain modifications induced in the fibrinogen structure by HOCl partly differed from those induced by ozone [43].

In the presence of nitrite (NO_2_^−^) at the inflammation site, the aforementioned reaction proceeds to form a nitration agent, namely peroxynitrite (PN). A similar effect (i.e., oxidation and nitration) can be obtained using 3-morpholidisydnonimine (SIN-1) *in vitro* [4]. Fibrinogen nitration was noticed in patients with coronary artery disease [44], arterial [4] and venous [3] thrombotic disease, thrombus formation [45] and its progression during atherosclerosis [46,47], in patients with acute respiratory distress syndrome [48], cancer [7], and end-stage renal disease [49]. It was observed [4] that exposure of fibrinogen to SIN-1 decreases the lag phase of fibrin polymerization, increases dynamics of platelet aggregation, accelerates factor XIII cross-linking, and produces clots with the thinner twisted fibers and larger pores. Clots made of thinner fibrin fibers were also observed [33], although smaller pores were majorly reported. When exposed to PN [37,50], the Aα chain of fibrinogen, and particularly its αC domain, is more prone to nitration than the others chains. Exposure to PN also increases number of nitrotyrosine, dityrosine, and carbonyl groups and leads to cross-linking of Aα chains [37].

Malondialdehyde (MDA) is a reactive carbonyl compound that is released during membrane lipid oxidation and peroxidation and as a by-product of prostaglandin and thromboxane synthesis [51]. Oxidation of plasma lipids increases the risk of cardiovascular diseases [35]. Although MDA is associated with numerous pathological processes such as diabetes, cardiovascular diseases, particularly atherosclerosis, and cancer [52,53], the data describing its effect on fibrinogen are scarce. It was demonstrated [54] that the amount of MDA in blood positively correlates with the amount of fibrinogen. MDA reduces the speed of fibrin polymerization [40] and the clot made of fibrin modified by MDA has thinner fibres and smaller pores [33,40]. MDA reacts with protein amino acid residues resulting in the inter/intracrosslinking of proteins or formation of carbonyl groups in proteins [55].

To the best of our knowledge, only two studies use molecular dynamics (MD) simulations to describe the effect of PTMs on fibrinogen structure. Burney et al. [56] reported that oxidation of AαM476 shifts the equilibrium between open and closed conformation of the αC domain in favor of the former one and that oxidation of BβM367 does not influence the behavior of fibrinogen. Oxidation of AαM476 was also studied by Pederson et al. [57] who reported that this amino acid is necessary for αC domain dimerization and that its oxidation makes dimerization energetically unfavorable in comparison with the native structure.

The presented work is an extension of our previous work [33] where the effect of PTM on platelet dynamic adhesion and fibrin network architecture was studied. Here, we used mass spectrometry (MS) to identify the positions and nature of PTMs induced *in vitro* to fibrinogen by NaOCl (that in solution dissociate), MDA, and SIN-1. These reagents mimic the oxidative stress related to various diseases, such as inflammation [41], thrombotic diseases [3,4], atherosclerosis [47], and diabetes [58]. We used molecular dynamic simulations to study the impact of selected modifications on the fibrinogen secondary structure at an atomistic level. Electron microscopy was used to vizualize changes in fibrin clot structure on mesoscopic scale. In order to support the findings obtained from *in vitro* modified fibrinogen, simulations of additional PTMs detected in fibrinogen of septic patients were added to the study.

## Materials and methods

### Fibrinogen modification

Fibrinogen solution was prepared from lyophilized human fibrinogen (Sigma-Aldrich, Prague, Czech Republic) by dissolving it in deionised water. Concentration of fibrinogen was determined spectrophotometrically at 278 nm using an extinction coefficient 15.1 for 10 mg/ml solution. Working solution of fibrinogen was adjusted to 4 mg/ml by using phosphate buffer saline (PBS; 137 M NaCl, 2.7 mM KCl, 8 mM Na_2_HPO_4_∙12 H_2_O, 1.5 mM KH_2_PO_4_, and pH 7.4).

NaOCl (Sigma Aldrich, Prague, Czech Republic) was prepared by diluting the stock solution with PBS and its concentration was determined spectrophotometrically (ε_290_ = 350 M^−1^·cm^−1^) [59]. For fibrinogen modification by NaOCl (1.25 mM), the incubation time was 20 min at 37 °C.

MDA was prepared by acid hydrolysis of 1,1,3,3-tetra methoxypropane (Sigma-Aldrich, Prague, Czech Republic) and the concentration was determined spectrometrically (ε_245_ = 13,700 M^−1^·cm^−1^) [55]. Fibrinogen was further incubated in presence of MDA (10 mM) for 120 min at 37 °C in dark.

SIN-1 (Sigma-Aldrich) was dissolved in 50 mM potassium phosphate buffer at pH 5.0 [4]. Fibrinogen was incubated with SIN-1 (0.1 mM) for 60 min with vortexing after every 10 min of incubation at 37 °C. The control samples were exposed to similar conditions as the modified samples but without modification species. After incubation, fibrinogen was purified by centrifugal gel filtration (Sephadex G-25 superfine; Pharmacia, Uppsala, Sweden). Protein concentration in the eluate was estimated using Bradford protein assay.

Fibrinogen of septic patients was purified from citrated plasma.

### Ethics statement

All samples were obtained and analyzed in accordance with the regulations of the Ethics Committee of the Central Military University Hospital Prague, Czech Republic (Reference Number: 108/8-91/2015-UVN) and the Ethics Committee on Clinical Research of the Institute of Hematology and Blood Transfusion, Prague, Czech Republic (Reference Number: 12/02/2016). Prior to enrollment in the study, written informed consent was obtained from each subject. All data were analyzed anonymously. The study was carried out in accordance with the International Ethical Guidelines and the Declaration of Helsinki.

### Mass spectrometry

We used mass spectrometry, that is often used for characterization of biomolecules [60–62], for detection of PTMs of fibrinogen. Samples of modified, control, native and patient fibrinogen were prepared by trypsin digestion. Briefly, after electrophoresis, the fibrinogen chains were cut out from gel in approximately 1 mm^3^ pieces. Destaining was performed by incubation with 0.1 M NH_4_HCO_3_/acetonitrile (1:1) and acetonitrile. Rehydratation was carried out by adding 0.1 M NH_4_HCO_3_. Tris(2-carboxyethyl)phosphine (50 mM) was used as the reduction reagent; whereas, 2-iodoacetamide (55 mM) was used as an alkylating agent. Trypsin digestion (12.5 ng/ml in 25 mM NH_4_HCO_3_) was performed with constant shaking for 16 h at 37 °C. Reaction was hindered by adding 50% acetonitrile/0.1% formic acid. Lyofilisated samples were stored at −80 °C.

For identifying the modified amino acid residue, HCT ultra ion-trap mass spectrometer with nanoelectrospray ionization, coupled to an UltiMate 3000 nanoLC system, was used. Data analyses were performed by The esquireControl v6.2 software for data acquisition, DataAnalysis v4.0 for data processing, and BioTools v3.2 (all Bruker Daltonics), collectively with MASCOT v2.2 (Matrix Science, London, UK) for database searching. For each environment, at least three independent samples were prepared. A PTM is considered to be a consequence of the reagent if it was not observed in the control or native fibrinogen. PTM was considered when occurred at least in one sample.

### Scanning electron microscopy

Fibrin net architecture was studied by scanning electron microscopy (Mira 3 LMH, Tescan Orsay Holding, a.s., Brno, Czech Republic). Thrombin (final concentration 2 NIH U/ml) was added to both modified and control fibrinogen and incubated at room temperature for 3 h. The networks were fixated by 4% formaldehyde and were washed with PBS and water and subsequently dehydrated with a series of water–ethanol solutions with increasing ethanol concentration (0%, 25%, 50%, 75%, and 100%). Eventually, the samples were dried using the CO_2_ critical point method (Balzers CPD 010) and coated with 4 nm thick platinum by sputtering (Balzers SCD 050). Images were evaluated by ImageJ data analysis software (http://rsbweb.nih.gov/ij/) [63]. Fiber thickness and average number of fibrin fibers per 1 micrometer square of fibrin clot were determined by analyzing five different images captured from two independently prepared samples of both, modified and control fibrinogen. Fiber thickness was determined as mean ± SD from 200 values (20 measurements per image). Average number of fibers per 1 micrometer square was determined as mean ± SD from 10 images. The significance of differences was evaluated using student’s t-test. *P* values less than 0.05 (two-sided) were considered as statistically significant.

### Molecular dynamics simulations

Fibrinogen molecule contains 2964 amino acids what makes its multiple atomistic simulations unfeasible to us because of their computational demands. Instead of simulating the whole fibrinogen molecule we focused on MD simulation of fibrinogen regions important for the conversion of fibrinogen to fibrin or important for fibrinogen stability and certain physiological functions. Initial coordinates of fibrinogen used for MD simulations were obtained from crystal structure 3GHG [21]. To describe an effect of PTMs on the γ-nodule of fibrinogen, amino acids 148–394 from the C chain (numbering of amino acid residues and designation of chains follows the notion used for the 3GHG structure for both systems) and the corresponding Ca^2+^ ion, were used. For modeling the central part of the coiled-coil connector of fibrinogen amino acids 70–126 of the A chain, 101–157 of the B chain, and 47–97 of the C chain, were used (S1 Fig). Selected PTMs were introduced into the structure by Vienna-PTM 2.0 [64]. Each PTM was treated in an individual simulation and its behavior was compared with the simulation of wild type (WT) system. Notion of the amino acids for the mature form of fibrinogen chains (i.e. without transit peptide) is used in the entire article.

MD simulations were performed in Gromacs 5.1.1 [65] with Gromos 54a7 [66] force field that was recently extended for parameters for posttranslationally modified amino acids [67]. Production simulations with a time-step of 2 fs were performed for 100 ns, with the last 25 ns used for analyzing the coiled-coil connector systems and the last 50 ns of 250 ns simulation was used for analyzing the simulations of the γ-nodule. Simulations of the fully hydrated protein were performed at a physiological temperature of 37 °C and at atmospheric pressure. See S1 Appendix for detailed description of simulation protocol.

Frames taken every 250 ps were used for analyses performed by standard tools of Gromacs. Secondary structure was described by DSSP [68] as implemented in Gromacs.

Ramachandran plot was computed by Procheck [69] from the last frame of simulation, whose geometry was optimized by steepest descent algorithm as implemented in Gromacs.

Sequence analysis was performed on 87 mammalian protein sequences of the γY68–γM78 region (notion according to human mature γ chain of fibrinogen) of the fibrinogen γ chain, those were downloaded from the NIH database [32] using keyword FGG. Only one splicing variant was used for each species (both splicing variants of the γ chain of fibrinogen are identical in the region of interest). Sequences were aligned by ClustalX [70]. Sequence logo was created by WebLogo web server [71] and adjusted manualy (insertion in the sequence of European hedgehog, *Erinaceus europaeus*) in Inkscape.

## Results

### Mass spectrometry

**Fig 1:**
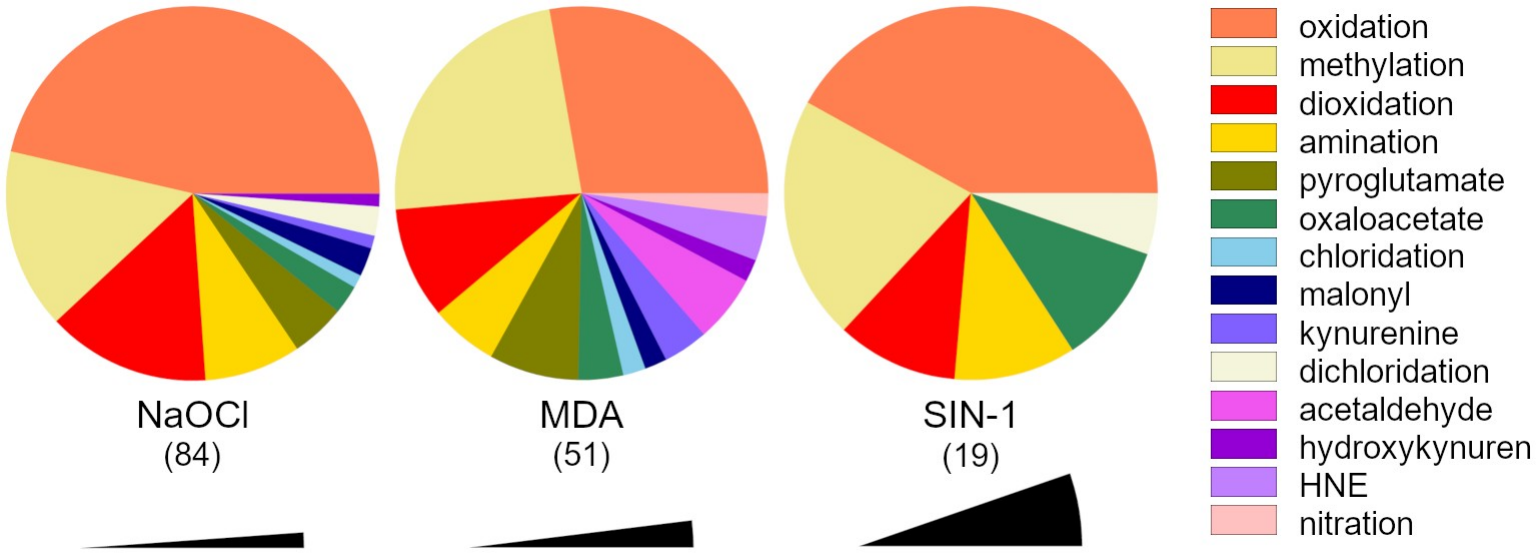
Proportion of PTMs that were induced into fibrinogen by the given reagent. Numbers below the abbreviations of the reagent names indicate the amount of unique PTMs. Black sectors represent size of one PTM in the graph.

The reagents modified 154 amino acids (see Fig 1 and S1 Table). Oxidation was the most frequent PTM transforming 61 residues, followed by methylation (29), dioxidation (19) and amination (12). Reagents induced pyroglutamate (8), oxaloacetate (6) acetyllysine, kynurenine, dichlorotyrosyl (each 3), chlorotyrosyl, docosahexaenoyl lysyl (HNE), hydroxykynurenine, malonylseryl (each 2), malonylcystyl and nitrotyrosyl (both 1) into fibrinogen structure.

The PTM count increased with the strength of the reagent used. NaOCl induced 84 PTMs, MDA 51, and SIN-1 19. The nature of PTMs induced into the fibrinogen by different reagents differed as well. MDA did not dichloridate any amino acid residue; however, it is the only reagent inducing acetaldehyde, nitro, and HNE groups into the fibrinogen structure. Reaction of fibrinogen with SIN-1 did not further induce any pyroglutamate, kynurenine, hydroxykynurenine, chloration, and malonylation into the protein.

The PTM count differed among the fibrinogen chains. Bβ chain was the most frequent target of the reagents. There were 35 PTMs induced into Bβ chain by both NaOCl and MDA and 12 PTMs by SIN-1. A similar number of PTMs was induced by the reagents into Aα chain (26 PTMs by NaOCl, 8 PTMs by MDA, 3 PTMs by SIN-1) and into γ chain (23 PTMs by NaOCl, 8 PTMs by MDA and 4 PTMs by SIN-1).

Different amino acids were modified with distinct frequencies. Lysine was the most commonly modified amino acid (26 residues) followed by tyrosine and tryptophan (both 22 residues). No PTMs were detected in alanine, glycine, isoleucine, leucine, methionine, glutamine, threonine, and valine.

Simulating all detected PTMs is far beyond our computational possibilities. For this reason, we chose several PTMs at the functional sites of fibrinogen as described in [32]. Namely, PTMs at positions BβK122 (oxidation by NaOCl and SIN-1 to allysine, Bβ(Ox)K122 (for chemical formulae of modified amino acids see S2 Fig) and acetaldehydation by MDA to N6-acetyllysine, Bβ(Ac)K122), BβK133 (oxidation by NaOCl to allysine, Bβ(Ox)K133; acetaldehydation by MDA to N6-acetyllysine, Bβ(Ac)K133) and γK58 (oxidation by NaOCl to allysine, γ(Ox)K58) are the N-terminal residues of peptide bonds cleaved by plasmin. Hementin cleaves peptide bonds containing BβK130 (acetaldehydation by MDA to N6-acetyllysine, Bβ(Ac)K130) and γP76 (oxidation by NaOCl to glutamic acid, γ(Ox)P76E) as a N-terminal amino acid residue of the cleavaged peptide bond. Amino acid residues γR375 (oxidation by NaOCl to citrulline, γ(Ox)R375), γK380 (oxidation by NaOCl to allysine, γ(Ox)K380) and γK381 (oxidation by NaOCl to allysine, γ(Ox)K381) are within the a-hole, a binding site for N-terminus of an α chain of fibrin during polymerization.

In-depth analysis of fibrinogen PTMs in samples from septic patients is reported elsewhere [Štikarová et al, in preparation]. Here, we report only some MD simulations of PTMs whose manifestation supports hypotheses and findings drawn from simulations of *in vitro*-induced PTMs. The reported PTMs are oxidation of AαM91 to methionine sulfoxide (Aα(Ox)M91), that was performed to see the impact of PTMs in Aα chain on behaviour of the coiled-coil connector. Oxidation of BβN140 to aspartic acid (Bβ(Ox)N140) occurs in the region of Bβ chain that was transformed to π-helix in simulations describing *in vitro*-induced PTMs and oxidation of γP76 to pyroglutamic acid (γ(Ox)P76PGA) was examined to confirm the need of the amino acid with secondary amine for preservance of the loop region with the γ chain of coiled-coil connector.

### Scanning electron microscopy

**Fig 2:**
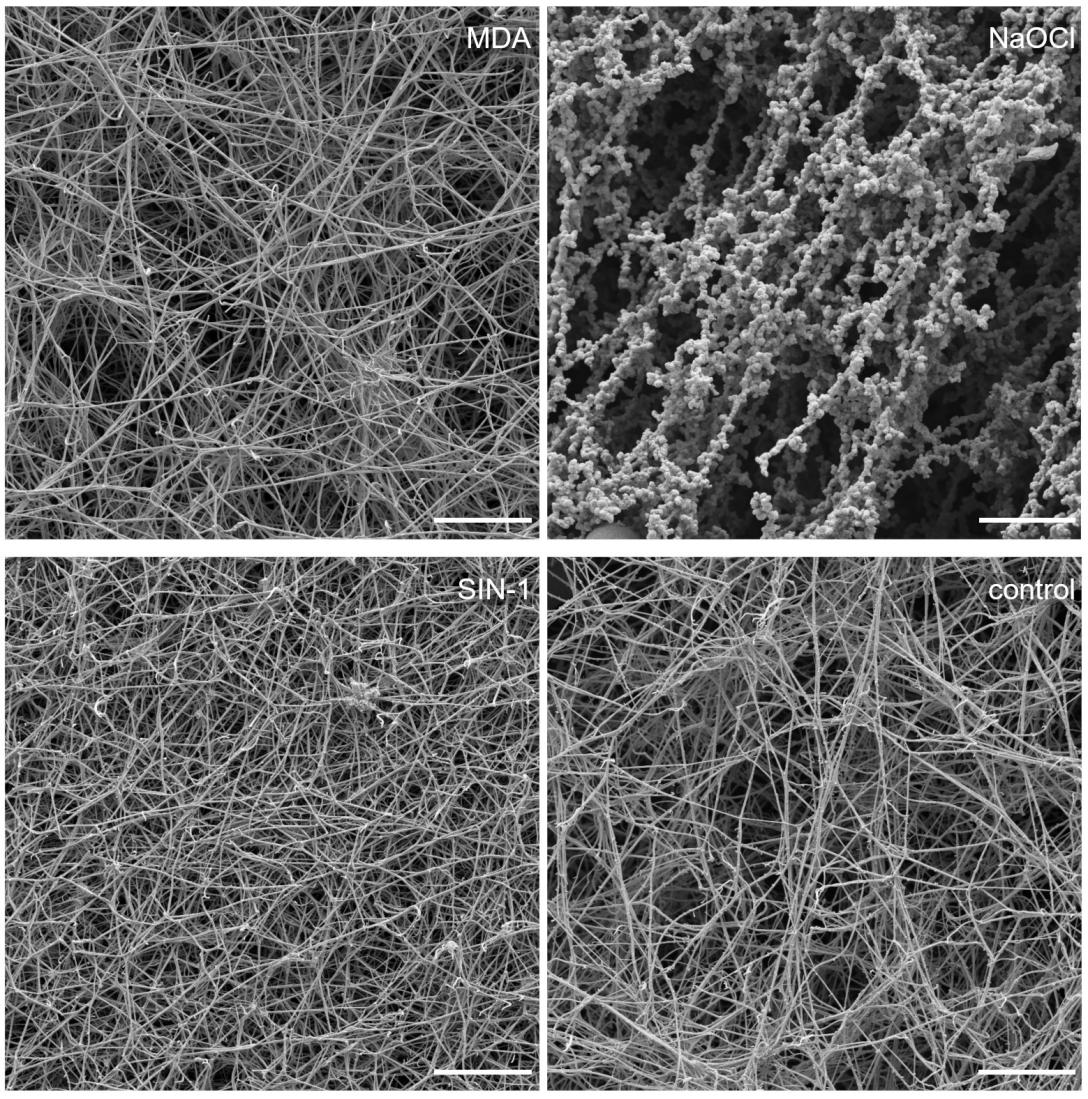
Representative images captured by scanning electron microscope of fibrin net made from modified and control fibrinogen. The scale bar is 10 μm.

Fibrinogen modified by all three reagents formed a fibrin clot. The most distinguished net was formed from fibrinogen modified by NaOCl (Fig 2). Very few strands were observed with visible knobs, formed by very thin fibers, around them. Significantly, fewer and thicker strands were observed in the fibrin net from MDA modified fibrinogen in comparison with the control fibrinogen. Significantly thinner strands were detected in the nets formed from SIN-1 modified fibrinogen compared to the control fibrinogen (Table 1).

**Table 1:** Fiber thickness and average number of fibrin fibers per 1 μm^2^ of fibrin clot.

Fibers thickness (nm)	Control	NaOCl		MDA	SIN-1
		Knobs	Fibers		
	129.4 ± 87.6	634.7 ± 271.7	40.9 ± 12.1	183.7 ± 77.4	100.1 ± 51.3
Average no. of fibers strands per field (1 μm^2^)	10.5 ± 4.4	1.9 ± 0.58		5.4 ± 1.3	10.7 ± 1.8

### Molecular dynamics simulations

#### Posttranslational modifications in the coiled-coil connector of fibrinogen

Root mean square deviation (RMSD) of atomistic positions with respect to their initial positions can be interpreted as a measure of divergence of the analyzed structure from the initial structure. Here, we compared averages of RMSD of C_α_ carbons over the last 25 ns of the simulations of modified fibrinogen and WT (Table 2; S3 Fig). RMSD of the Aα(Ox)M91 was much higher than those of the others systems (by 61% of the WT value) what can be interpreted as the geometry of this structure differed from the geometry of the WT more than the geometries of the others systems. RMSD of Bβ(Ox)K122, Bβ(Ac)K130 and Bβ(Ox)N140 was within 5% of the WT value indicating minor structural alternations from the WT geometry. RMSD of the others systems was within 9–20% of the WT value.

**Table 2:** Comparison of averages of RMSD of C_α_ carbons over the last 25 ns of MD simulations. Systems whose RMSD did not equilibrated within the 100 ns MD simulation (S3 Fig) are marked by an asterisk (*).

	RMSD [nm]			
	system	Aα	Bβ	γ
**WT**	0.46 ± 0.04	0.30 ± 0.05	0.33 ± 0.03	0.44 ± 0.04
**Bβ(Ac)K122**	0.45 ± 0.02	0.25 ± 0.03	0.42 ± 0.03	0.38 ± 0.03
**Bβ(Ox)K122**	0.50 ± 0.03	0.25 ± 0.03	0.47 ± 0.05	0.49 ± 0.05
Bβ(Ac)K130*	0.44 ± 0.05	0.27 ± 0.07	0.36 ± 0.07	0.39 ± 0.02
**Bβ(Ac)K133**	0.52 ± 0.04	0.30 ± 0.03	0.53 ± 0.06	0.42 ± 0.04
Bβ(Ox)K133*	0.54 ± 0.06	0.53 ± 0.06	0.38 ± 0.05	0.49 ± 0.04
**γ(Ox)K58**	0.55 ± 0.04	0.38 ± 0.04	0.39 ± 0.06	0.43 ± 0.03
**γ(Ox)P76E**	0.52 ± 0.05	0.38 ± 0.06	0.39 ± 0.04	0.46 ± 0.04
**Aα(Ox)M91**	0.74 ± 0.06	0.66 + 0.09	0.57 ± 0.06	0.56 ± 0.06
Bβ(Ox)N140*	0.47 ± 0.05	0.35 ± 0.04	0.34 ± 0.05	0.52 ± 0.06
**γ(Ox)P76PGA**	0.51 ± 0.03	0.27 ± 0.04	0.38 ± 0.035	0.50 ± 0.02

We compared averages over the last 25 ns of RMSD of C_α_ carbons for individual chains of fibrinogen. This comparison revealed that RMSD differed in various extend over chains and among systems. This can be interpreted as the PTM influence structure of fibrinogen chains in various extent.

When RMSD of the C_α_ carbons of protein converges to a certain value, that differs among various molecules, the geometry of the system reached local equilibrium. In our simulations of the coiled-coil connector of fibrinogen, all systems reached equilibrium but for Bβ(Ac)K130, Bβ(Ox)K133 and Bβ(Ox)K140 whose development is ongoing. We must bear this in mind when interpreting the results.

Radius of gyration characterizes the measure of compactness of a structure. Averages of radius of gyration of the C_α_ carbons over the last 25 ns (Table 3, S4 Fig) are within 5% of the average radius of gyration of the WT, what can be interpreted as PTMs do not induce split of the coiled-coil connector (nor its collapse). Concerning the radii of gyration of individual chains, the increased value for the Bβ chain of the Bβ(Ac)K122 can be explained by unfolding of the α-helix (see below) and the increased values of radius of gyration of C_α_ carbons of the γ chain for some systems (Bβ(Ac)K122, Bβ(Ox)K122, Bβ(Ac)K133, γ(Ox)K58, Aα(Ox)M91) resulted from the dynamics of the unfolded part of the γ chain (see below).

**Table 3:** Comparison of averages of radius of gyration of C_α_ carbons over the last 25 ns of MD simulations of coiled-coil connector.

	Radius of gyration [nm]			
	system	Aα	Bβ	γ
**WT**	2.34 ± 0.03	2.42 ± 0.02	2.38 ± 0.04	2.03 ± 0.05
**Bβ(Ac)K122**	2.43 ± 0.02	2.44 ± 0.02	2.51 ± 0.03	2.22 ± 0.04
**Bβ(Ox)K122**	2.38 ± 0.03	2.42 ± 0.02	2.42 ± 0.03	2.19 ± 0.05
**Bβ(Ac)K130**	2.36 ± 0.02	2.42 ± 0.03	2.43 ± 0.03	2.12 ± 0.02
**Bβ(Ac)K133**	2.44 ± 0.02	2.45 ± 0.02	2.49 ± 0.04	2.24 ± 0.04
**Bβ(Ox)K133**	2.35 ± 0.03	2.37 ± 0.03	2.47 ± 0.03	2.10 ± 0.04
**γ(Ox)K58**	2.36 ± 0.02	2.39 ± 0.02	2.41 ± 0.04	2.16 ± 0.04
**γ(Ox)P76E**	2.33 ± 0.02	2.37 ± 0.03	2.45 ± 0.03	2.08 ± 0.03
**Aα(Ox)M91**	2.36 ± 0.05	2.36 ± 0.06	2.37 ± 0.04	2.27 ± 0.05
**Bβ(Ox)N140**	2.32 ± 0.04	2.40 ± 0.04	2.46 ± 0.04	1.94 ± 0.03
**γ(Ox)P76PGA**	2.36 ± 0.02	2.42 ± 0.03	2.42 ± 0.03	2.11 ± 0.03

Root mean square fluctuation (RMSF) characterizes a deviation of particle position from initial structure over time. Here, we computed RMSF for C_α_ carbons over the last 25 ns of MD simulation (S5 Fig) to see, if some regions of fibrinogen coiled-coil connector are more prone to structural changes than the others. The major changes of RMSF of modified systems in comparison with the WT were later assigned to regions, where α-helices of the coiled-coil connector change their secondary structure. Further variations are recognized within the unfolded part of the γ chain. Because of correlation of changes in RMSF and changes in secondary structure, the discussion of RMSF is incorporated into the section describing secondary structure of the coiled-coil connector and concerns only those systems with RMSF alternations from the WT.

DSSP algorithm, that is based on Kabsch-Sander classification of protein secondary structure [72], characterizes secondary structure elements according to the defined patterns of hydrogen bonds within a protein.

**Fig 3:**
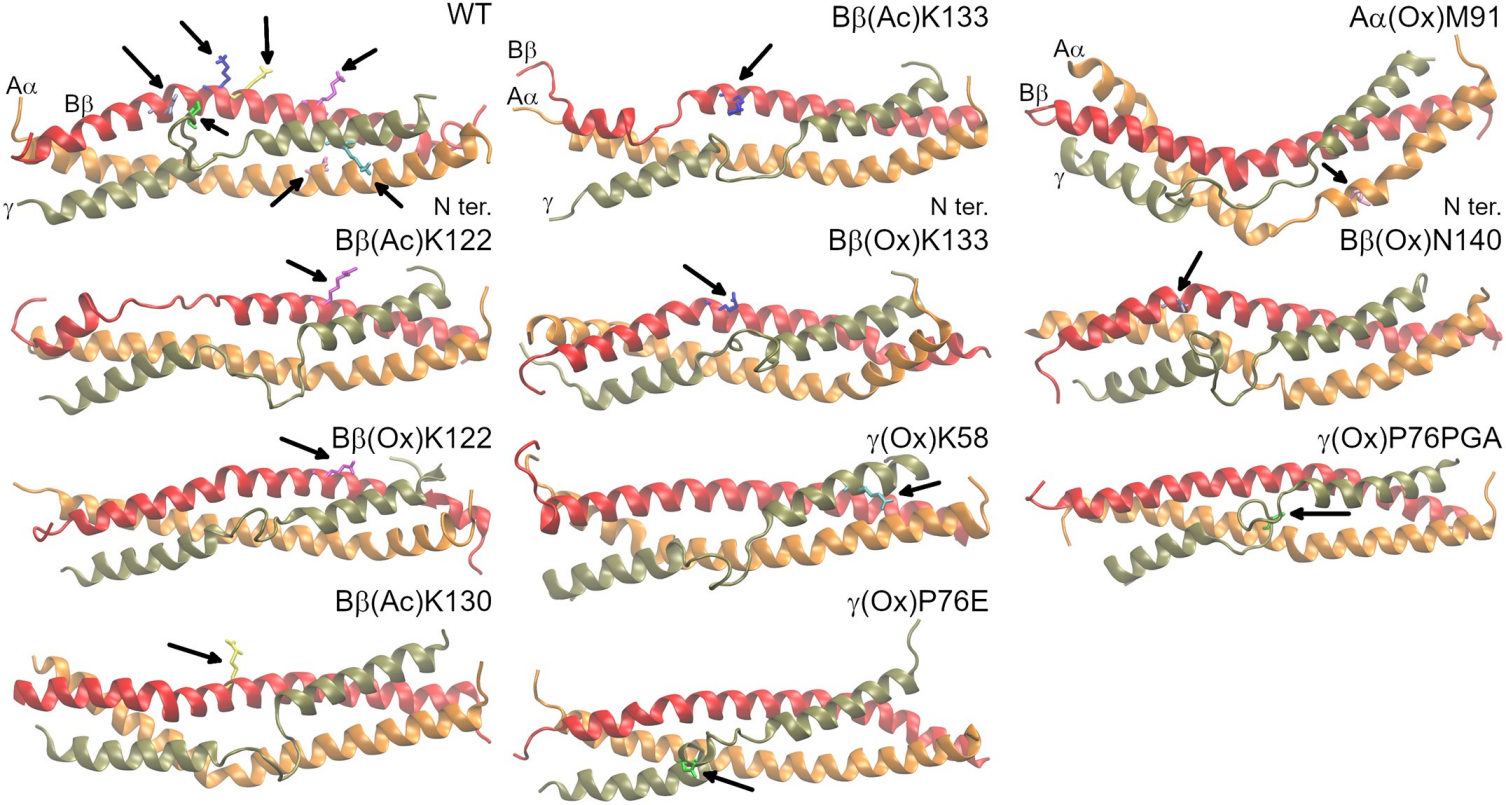
Comparison of the resultant frame of the simulation for systems with PTM in the coiled-coil connector of fibrinogen with WT. Aα chain is presented in orange, Bβ chain in red, and γ chain in brown. Amino acids including their PTMs are highlighted in the following colors: AαM91, pink; BβK122, magenta; BβK130, yellow; BβK133, blue; BβN140, violet; γK58, cyan; and γP76, green.

Analysis of secondary structure revealed, that WT of the coiled-coil connector of fibrinogen (Fig 3 and S6 Fig) preserved its structure during 100 ns MD simulation, but for occasional (occurring in 68% of frames analyzed since 47.5 ns) π-helical disturbancies of the Bβ chain involving amino acids BβN135–BβV139. Other transient π-helices formed from amino acids BβK130–BβD134 resp. BβN140–BβS144 were formed between 30.5 and 34.5 ns (50% frames) resp. between 19.5 and 28.75 ns (69% frames) and between 34.75 and 38 ns (69% frames). The most significant feature of RMSF of this system was an increase of RMSF in the middle of the γ chain. It represents the flexible unfolded part of the γ chain.

Formation of a π-helix (observed in 91% of analyzed frames since 13.5 ns) made by amino acids BβQ131–BβN135 was the most prominent structural change in the system describing oxidation of BβK122 to allysine, Bβ(Ox)K122 (Fig 3 and S6 Fig). Secondary structure of Aα and γ chains remained unaltered.

Acetaldehydation of BβK122, Bβ(Ac)K122 (Fig 3 and S6 Fig), resulted into unfolding of a segment of the α-helix formed by amino acids BβD134–BβE141 since beginning of the simulation. This disturbance was followed by one-turned π-helix (BβY142–BβL146) on its C-terminus that appeared at 44.75 ns and since it was presented in 93% of frames. These disturbancies were manifested by increased RMSF in comparison with WT. They occured at different positions than the π-helix in the WT and thus, they do not average out. Analogically, the π-helix in the WT structure increased the RMSF of amino acids BβQ131–BβN135 of the WT.

Simulation describing the impact of acetaldehydation of BβK130, Bβ(Ac)K130 (Fig 3 and S6 Fig), did not reach local equilibrium of RMSD of C_α_ carbons within 100 ns (see S3 Fig). This indicates that the system development is in process. At the end of the simulation, all chains preserved their initial structure, but for the π-helix made of amino acids BβV138–BβY142, that appeared at 80.75 ns and since it was recognized in 86% of frames. Increase of RMSF for amino acids AαS99–AαN106 indicates some instability, that did not precluded at the level of secondary structure yet. RMSF of the unfolded region of the γ chain was decreased in comparison with WT. This points to stabilization of the unfolded region.

Oxidation of BβK133 to allysine, Bβ(Ox)K133 (Fig 3 and S6 Fig), is the other system, whose RMSD did not reached equilibrium within 100 ns MD simulation and thus it still undergoes development. Bβ(Ox)K133 did not influence secondary structure of the coiled-coil connector of fibrinogen on the level of DSSP. Analysis of RMSF revealed increased flexibility between AαF98–AαT107 that is demonstrated by transitionally disturbed α-helical structure for amino acids AαR104 (α-helix in 80% of frames over the whole trajectory). Analysis of RMSF of the γ chain showed wider unfolded region (γY66–γM78) and increased flexibility of the adjacent α-helix in comparison with RMSF of WT.

Acetaldehydation of BβK133, Bβ(Ac)K133 (Fig 3 and S6 Fig), disturbed the Bβ chain on fibrinogen between amino acids BβN137 and BβL146. Amino acids BβE141 and BβY142 did not adopted α-helical structure since beginning of the simulation. Since 57.25 ns the interuption in the α-helix extended in the N-terminal direction towards BβN137 not forming any prevalent secondary structure element. A turn formed by amino acids BβY142—BβS143 resp. BβS144 was recognized between 57.25 and 70.25 ns. Then it was transformed into π-helix containing amino acids BβY142—BβL146 that preserved till the end of simulation. This behavior is characterized by increase of RMSF at the C-terminus of the Bβ chain (BβN137—BβL151). RMSF of the unfolded region of the γ chain decreased in comparison with WT.

Oxidation of γK58 to allysine, γ(Ox)K58 (Fig 3 and S6 Fig), did not introduce any obvious alterations within the 100ns MD simulation. More in-depth insight revealed disintegration of the hydrogen bond connecting γallysine58 and γK62 starting from 25 ns. This behavior was not observed for the WT. Switches of the α-helix to turn in the Aα chain involving residues AαR110 and AαV111 were observed in 27% of analyzed frames since 13.75 ns. Chains Bβ and γ were unaltered by γ(Ox)K58.

Oxidation of γP76 (Fig 3 and S6 Fig) to glutamic acid, γ(Ox)P76E, resulted into formation of a short α-helix made of amino acids γE72–γN77 at 26 ns. This helix did not extend the already existing α-helix at the C-terminus of the simulated part of γ chain, which was linked to it by γM78 identified as a bend by DSSP. Furthermore, it unfolds at 61 ns and its refolding at 67.25 ns is connected with disturbancies in α-helices of Aα and Bβ chains. Namely, amino acids AαR95 and AαG96 switches to turn at 68 ns and at 76 ns π-helix containing amino acids AαL94—AαF98 is formed. A short π-helix made of amino acids AαL94–AαF98 was formed at 76.25 ns and was preceded by a turn made of AαR95 and Aα96 since 68 ns. An α-helix of the Bβ chain is corrupted between 67.75–76.5 ns in the region BβL100—BβA106 (π-helix and turn), between 81.5–86.5 ns and since 96.5 ns (π-helix made of BβS108—BβS112 for both interruptions). This behavior is manifested by increased RMSF of the Aα and Bβ chains and of N-terminal part of the γ chains, while RMSF of the unfolded region of the γ chain is decreased in comparison with the RMSF of WT (S5 Fig).

The most significant consequence of oxidation of AαM91 to methionine sulfoxide (Fig 3 and S6 Fig), Aα(Ox)M91, was disintegration of an α-helix of Aα chain involving AαR95—AαF98 that started at 53 ns. It was followed (since 54 ns) by a conversion of BβK130—BβD134 of the Bβ chain from α-helix to π-helix. Secondary structure of the γ chain remained unaltered. RMSF of Aα and Bβ chains is increased in the altered regions as is RMSF of the γ chain in N-terminal direction with respect to the unfolded region, whose RMSF is in comparison with WT decreased.

Oxidation of BβN140 to aspartic acid (Fig 3 and S6 Fig), Bβ(Ox)N140, is the last system that did not reached equilibrium within 100 ns simulation. This PTM destabilized Aα chain of fibrinogen. Starting at 9.5 ns, amino acids AαL94—AαS99 interchanged their conformation between 3_10_ helix (51% of the analyzed frames), turn (23%), π-helix (21%) and α-helix (5%). Another π-helix (AαV111—AαL115) was formed at 89.25 ns (observed at 86% of frames since 89.25 ns). Bβ and γ chain of fibrinogen remains unaltered by this PTM. Both of these disturbances were joined with increased RMSF. RMSF is further increased between amino acids BβQ131—BβV138 although this alternation was not shown on the secondary structure level.

Unlike its oxidation to glutamic acid, oxidation of γP76 to pyroglutamic acid (Fig 3 and S6 Fig), γ(Ox)P76PGA, did not fold the disordered region of the γ chain (amino acids γY68—γM78). This can be interpreted as a need of amino acids with the secondary amine for preserving the disordered loop within the γ chain of fibrinogen. Simulation further revealed a conversion of amino acids AαL94—AαF98 from the α-helix to π-helix since 82.75 ns (86% of analyzed frames since 82.75 ns). DSSP revealed extension of the unfolded part of the γ chain up to γA81, what was not observed in the other systems.

Analyses of secondary structure (S6 Fig and Table 4) further revealed that although the unfolded region of the γ chain was preserved in all systems its secondary structure content varies among systems. Amino acids of the unfolded region of the γ chain adopt coil, bend and turn (but for Aα(Ox)M91) although the number of amino acids adopting these structures was variable. Bβ(Ac)K130 and Bβ(Ox)N140 also exhibit some amino acids in 3_10_-helical conformation. As the development of both systems is ongoing it is questionable if 3_10_-helix is not exhibited only temporarily. As mentioned above, α-helix is formed in the γ(Ox)P76E system. Further work is being performed to characterize and understand behavior of the loop. This knowledge would enable characterization of an impact of PTMs to this region of fibrinogen coiled-coil connector more in-depth.

**Table 4:** Extent of the unfolded region of the γ chain (N- and C-terminal amino acids of the unfolded region) and secondary structure content (number of amino acids adopting given type of secondary structure) of the unfolded region of the γ chain averaged over the last 25 ns of simulation.

	extent	coil	bend	turn	α-helix	3_10_-helix
**WT**	γT67—γM78	8.0 ± 0.8	2.6 ± 0.8	1.4 ± 0.9		
**Bβ(Ac)K122**	γY68—γI79	8.4 ± 0.9	2.6 ± 0.8	1.0 ± 1.0		
**Bβ(Ox)K122**	γY68—γN77	6.6 ± 0.2	5.3 ± 0.8	3.0 ± 0.2		
**Bβ(Ac)K130**	γY68—γM78	5.8 ± 0.8	2.7 ± 1.1	2.4 ± 1.2		1.5 ± 0.7
**Bβ(Ac)K133**	γY68—γM78	7.3 ± 0.6	2.2 ± 0.8	1.5 ± 0.9		
**Bβ(Ox)K133**	γN69—γM78	6.0 ± 0.6	1.2 ± 0.8	2.8 ± 1.0		
**γ(Ox)K58**	γY68—γM78	6.8 ± 0.7	1.0 ± 0.5	3.2 ± 0.6		
**γ(Ox)P76E**	γY68—γM78	5.1 ± 1.2	4.0 ± 0.2	1.3 ± 1.8	4.2 ± 2.3	3.7 ± 1.1
**Aα(Ox)M91**	γT67—γM78	9.1 ± 0.8	2.9 ± 0.8			
**Bβ(Ox)N140**	γY68—γM78	6.6 ± 0.8	3.2 ± 0.8	1.2 ± 1.0		
**γ(Ox)P76PGA**	γY68—γA81	7.3 ± 1.3	3.8 ± 1.3	2.7 ± 0.9		0.2 ± 0.8

Analysis of the secondary structure (S6 Fig) revealed disturbancies of α-helices of Bβ and Aα chains by usually π-helices and turns. π-helices were at similar positions (BβK130—BβY142 resp. AαL94—AαF98) among systems (Table 5). In case the α-helix is disturbed by coil or turn, these secondary structure elements were located at the above mentioned positions and π-helices were formed latterly on the C-terminus of the disturbancy (Bβ(Ac)K122 and Bβ(Ac)K133). There is an α-helical region between (mostly) 3_10_-helical disturbancy and the π-helix in simulation of Bβ(Ox)N140. As the geometry of this system had not equilibrated yet, further development of these structures is expected. The disturbancies occurred either in Aα or (more frequently) in Bβ chain, but for Aα(Ox)M91 where they were in both Aα and Bβ chain and appeared within 1 ns.

**Table 5:** Summary of disturbances in α-helical structure of the coiled-coil connector and characterization of kinks joined with π-helices. Systems absent in the table preserved their α-helical structure uncorrupted.

	π-helix			other	
	position	appear [ns]	tilt [°]	position	appear [ns]
**WT**	BβN135—BβV139	47.5	35.6 ± 4.2		
**Bβ(Ac)K122**	BβY142—BβL146	44.75		Bβ134—BβE141	0
**Bβ(Ox)K122**	BβQ131—BβN135	13.5	23.8 ± 7.6		
**Bβ(Ac)K130**	BβV138—BβY142	80.75	28.5 ± 5.9		
**Bβ(Ac)K133**	BβY142—BβL146	70.25		BβN137—BβE141	0
**γ(Ox)P76E**	AαL94—AαF98	76.25	33.7 ± 8.5		
**Aα(Ox)M91**	BβK130—BβD134	54.0	29.0 ± 10.2	AαR95—AαF98	53.0
**Bβ(Ox)N140**	AαV111—AαL115	89.25		AαL94—AαS99	9.5
**γ(Ox)P76PGA**	AαL94—AαF98	82.75	24.0 ± 6.6		

π-helices introduces kinks into α-helical structures [73]. These kinks are obvious in the final frames of the simulations (Fig 3). We computed an angle between center of mass of three C_α_ carbons of N-terminal amino acids adopting α-helix, C_α_ carbons of amino acids forming the π-helix and three C-terminal C_α_ carbons forming α-helix (S7 Fig). This analysis revealed increase of this angle when π-helix is formed. We did not analysed systems, where π-helix is preceded by turn or coil.

In order to quantify the kink in α-helix, we computed averages of the kink angle (described above) since the π-helix appreared in the system (Table 5). The kinks are in range 24–36° for both Aα and Bβ chains.

#### Posttranslational modifications in the γ-nodule of fibrinogen

RMSD of C_α_ carbons equilibrated over the last 50 ns of the 250 ns MD simulation for WT and γ(Ox)K380, unlike γ(Ox)R375 and γ(Ox)K381 where the structural development is ongoing (S8 Fig). Geometry of the γ(Ox)K380 varied from the geometry of WT in smaller extent (average RMSD of C_α_ carbons over last 50 ns of simulation is 0.32 ± 0.013 nm for WT; 0.30 ± 0.014 nm for γ(Ox)K380) than those of γ(Ox)K381 (0.37 ± 0.026 nm) and γ(Ox)R375 (0.39 ± 0.021 nm). Systems did not collapsed nor split, as determined by analysis of radius of gyration (S9 Fig).

**Fig 4:**
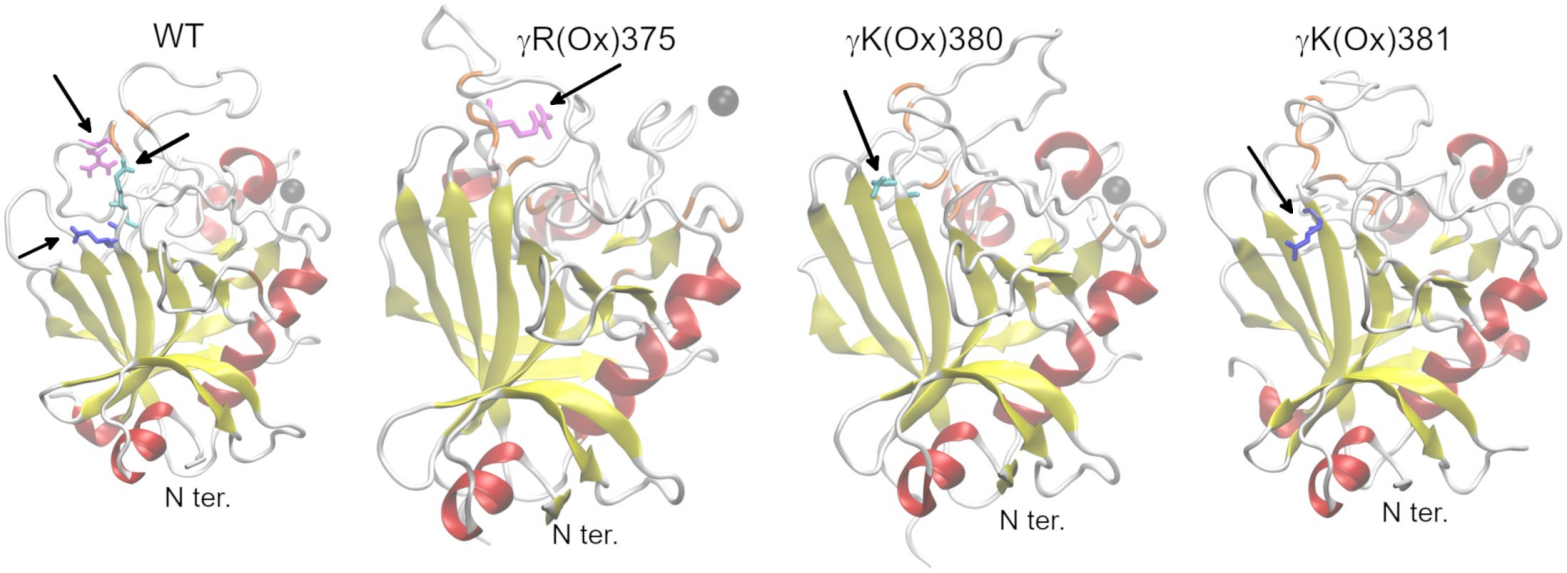
Comparison of the resultant frame of the simulation for systems with PTM in the C-terminal domain of the γ chain of fibrinogen with WT. α-helices, red; β-sheets, yellow; β-bridges, orange, loops and turns, white; Ca^2+^ ion, black; γR375, magenta; γK380, cyan; and γK381, blue.

During the 250 ns MD simulation of the γ-nodule of fibrinogen, WT preserved the architecture of the A subdomain (amino acids γV143–γW191) and of the B subdomain (amino acids γT192–γA286 and γK380–γL392 [28]; Fig 4 and S10 Fig). Apart from the two short α-helices and a β-sheet, the P subdomain (amino acids γG287 to γT379) comprised mainly unstructured regions, whose amino acids mutually interacted forming β-bridges. Ca^2+^ ion, necessary for appropriate polymerization of fibrin [74], preserved its position in the binding site (γD318, γD320, and γP322) over the whole course of the simulation.

Oxidation of the γK381, γ(Ox)K381, introduced only minor alternations into the structure of the γ-nodule (Fig 4 and S10 Fig) of fibrinogen that is present in the vicinity of the modified amino acid. Namely, certain disturbances were observed in the stability of the N-terminal α-helix of the P subdomain leading to its shortening by two C-terminal amino acids since 229 ns. The modification did not influence the Ca^2+^ binding.

Effect of γK380 oxidation, γ(Ox)K380, to the secondary structure of fibrinogen was negligible and Ca^2+^ ion remained at its binding site. A hydrogen bond network was observed in the WT linking γK380 with γK381 via γE251, whereas in the modified system, γK380 forms a hydrogen bond only with γY348.

Leaving the secondary structure of A and B subdomains of fibrinogen unaltered, oxidation of γR375, γ(Ox)R375, had considerable effect on the structure and behavior of the P subdomain. The C-terminal α-helix (γC326–γD330) in the P subdomain unfolded at 19 ns, and there was an antiparallel, two-stranded β-sheet formed at the C-terminus (γI386–γS378) of the P subdomain at 79 ns (Fig 4 and S10 Fig). The most significant event in the dynamics of this system was release of Ca^2+^ ion at 215 ns that had been preceded by destabilization of its binding site since 125 ns (S11 Fig). An extensive hydrogen bond network comprising modified γR375 was formed in the P subdomain that stabilized the C-terminal (S12 Fig).

Analysis of RMSF of C_α_ carbons of γ-nodule of fibrinogen over the last 50 ns of simulation revealed considerably increased RMSF between γG292 and γH307 for all modified fibrinogens in comparison with the WT structure (S13 Fig). This highlights the increased movement of the N-terminal region of the P subdomain including the α-helix.

## Discussion

Pathological PTMs of fibrinogen are observed in various diseases including cardiovascular diseases, neurodegeneration diseases, or cancer. These changes in the amino acid residues are attributed, inter alia, to oxidative stress reagents [9]. Effect of oxidative stress on fibrinogen was extensively studied mainly on the mesoscopic scale. To the best of our knowledge, only few studies have been performed on more detailed level using either MS to identify the position and nature of the given PTMs (e.g., [35,42,43]) or MD ([56,57]) to observe the early stages of fibrinogen structural alternations after the PTM induction. In this study, we combined MS and MD in order to describe the mechanism by which PTMs may influence the behavior of fibrinogen. Previously, we [33] reported that MDA, NaOCl, and SIN-1 have various effects on fibrinogen functions, such as fibrin polymerization or interaction with platelets. These differences may be explained by various strength and mode of reagent action, as described earlier [75]. These observations did not support the statement [76] that all oxidizing reagents have considerably similar effect on fibrinogen.

We identified 154 PTMs as a result of fibrinogen reaction with reagent, most of which (35 PTMs for both NaOCl and MDA and 12 PTMs for SIN-1) were on the Bβ chain of fibrinogen. Similar PTM count was observed in Aα chain (26 by NaOCl, 8 by MDA, and 3 by SIN-1) and in γ chain (23 by NaOCl, 8 by MDA and 4 by SIN-1). In contrast, Yurina et al. [42] and Nowak et al. [37] found that higher amounts of PTMs induced by OCl^−^ in the Aα chain of human fibrinogen than those in the Bβ and γ chains. This disagreement could be explained by a different technique for PTM detection (we use MS, Nowak et al use combination of gel electrophoresis and Western blot with anti-nitrotyrosine and anti-DNP antibodies). Ambiguities among Yurina’s et al. and our work can be ascribed to a different way of both fibrinogen and hypochlorite preparation and nonidentical conditions under which PTMs were introduced.

Only six amino acid residues modified by OCl^−^ were found in both Yurina’s [42] study and the present work, namely AαD477, BβP216, BβW293, γW76, γF226 (dioxidated), and γH340. Furthermore, Yurina et al. [42] noticed BβD154 to be oxidized by OCl^−^. We found that BβD154 is modified by both, MDA and SIN-1, but not by NaOCl. To explain the differences in amino acids modified by OCl^−^, one must consider the different experimental setup.

We have performed MD simulations to see alterations, if any, in the structure of modified fibrinogen at the atomistic level. Such alternations may be joined with an altered structure of fibrinogen as well as may have an impact on fibrinogen interactions with other molecules and other fibrinogen properties. The inability of circular dichroism to detect changes in the fibrinogen secondary structure ([4,35,40]) may be caused by the amount of secondary structure changes that is beyond the limit of detection by circular dichroism.

Interpreting results of MD simulations, one has to consider that the simulations were performed only for 100 or 250 ns, whereas changes in the secondary structure occurred at the timescales of (tens of) microseconds. Therefore, the simulations presented here capture only the initial step in the conversion of the WT structure. Thus, the length of simulations is satisfactory. Comparison of different systems reveals that regions that were not in contact with the modified site behave in almost identical way. This observation demonstrates that different behavior among the systems is not an artifact of simulation or force field, but it is a consequence of the introduced PTM.

To make the simulations feasible, only fragments of the fibrinogen rather than the whole molecules were examined. This approach may introduce some artifact at the edges of the molecules; thus, the behavior of N- and C-terminal regions of simulated protein fragments remained uninterpreted.

Explaining the changes in fibrin clot structure in the content of result of our MD simulations, one must be aware of the observed architecture of fibrin clot is a result of all PTMs within the sample, not only of those characterized in this work. Fibrin fibers contain many mutually interacting fibrin monomers. Our model does not capture all such interactions. It describes an impact of the selected PTM on the secondary structure and treats fibrinogen as an isolated molecule in water. Calculations of more complex systems than presented are beyond our computational capacities. MD simulations give us a hint, how the PTM could alter secondary structure of fibrinogen in its vicinity and allow us to hypothesize, how this change could influence the structure of fibrin clot.

Each PTM is treated in separate system in MD simulations. Although never reported explicitly, the presence of more PTMs in one fibrinogen molecule cannot be excluded. If two or more PTMs were in the proximity of each other, their impact on fibrinogen could be different then if they were treated in isolated systems.

Simulation of the coiled-coil connector revealed a change in the protein structure from α-helix to either π-helix, turn or coil, which occurs in the vicinity of amino acids BβE140 and AαR95, with the exact position depending on the system (amino acids BβK130 to BβK146 and AαL94 to AαF98 + AαV111 to AαL115). Although no changes were observed in the systems Bβ(Ox)K58 and Bβ(Ox)K133, presence of alternations seems to be proper to the coiled-coil connector. π-helices are known to occur at or close to the binding sites of protein ligands as they destabilize the rigid structure of α-helices allowing ligand binding [77]. There are cleavage sites for hementin (peptide bonds AαN102—AαN103 and BβK130—BβQ131) and for plasmin (peptide bonds AαR104—AαD105 and BβK133—BβD134) [32] close to the region where π-helices appear in our simulations. As π-helices are absent in the input crystal structure (3GHG) and appear during MD simulation hence, we assume that these π-helices originating from the internal dynamics of the coiled-coil connector region of fibrinogen.

Kohler et al [78], who described the behavior of fibrinogen coiled-coil connector by more advanced MD simulations, reported bending of this region as well, although in bigger extend. The differences in the extent of bends can be ascribed to different system settings and force field used.

It seems likely that the bending of the coiled-coil connector is driven by the disordered region within the γ chain. Coiled-coil domains are stabilized by hydrophobic interactions among amino acids oriented toward the center of the coiled-coil domain, making a zipper-like structure [79]. Absence of hydrophobic amino acids of the γ chain, those adopt a disordered structure in region γY68–γM78, may destabilize the structure of the coiled-coil domain. As α-helices of the coiled-coil domain closely interact with each other, a kink within one chain would initialize bending of the others chains.

The γ chain forms a flexible loop by amino acids γY68–γM78. Crystal structure 3GHG shows that C-terminus of this loop is in close proximity of the Bβ chain (hydrogen bond between BβN140 and γN77) and thus mutual interactions of these chains are expected. We hypothesize that the internal dynamics of the loop enables interaction between Bβ and γ chains over the whole extent of the loop and over the corresponding region of the Bβ chain. Further simulations are conducted to verify this hypothesis as well as to characterize direct interactions between the unfolded part of the γ chain and the Aα chain. We further hypothesize that interactions among γ and Aα chains may be intermediated via interactions of γ and Bβ chains.

Our results revealed that PTMs may influence the dynamics of the unfolded region of the γ chain, whose interactions with especially the Bβ chain likely play a role in bending of the coiled-coil connector. Some PTMs also unfolded the α-helices of the Aα (Aα(Ox)M91) and Bβ (Bβ(Ac)K152 and Bβ(Ac)K133) chains. This finding may explain observation of Vadseth et al [4], that oxidation and nitration of fibrinogen decreases stiffness of fibrin clot.

Oxidation of γP76 to glutamic acid, but not to pyroglutamic acid, resulted into folding of amino acids γE72–γN77 to a short α-helix that was linked to the C-terminal part of the coiled-coil α-helix by a bend made of amino acids γM78 and γI79. In Ramachandran plot, there is γM78 located in the region typical for collagen triple helix (S14 Fig). A considerable shift in the ψ dihedral would be required to get this amino acid into the region of Ramachandran plot that is favored by right-handed α-helices. This is highly improbable and points to the preservance of the break in the α-helix. This may shorten the flexible unfolded part of the γ chain of fibrinogen, and thus, alter the frequency and extent of its encounters to the Bβ chain and eventually Aα chain.

**Fig 5:**
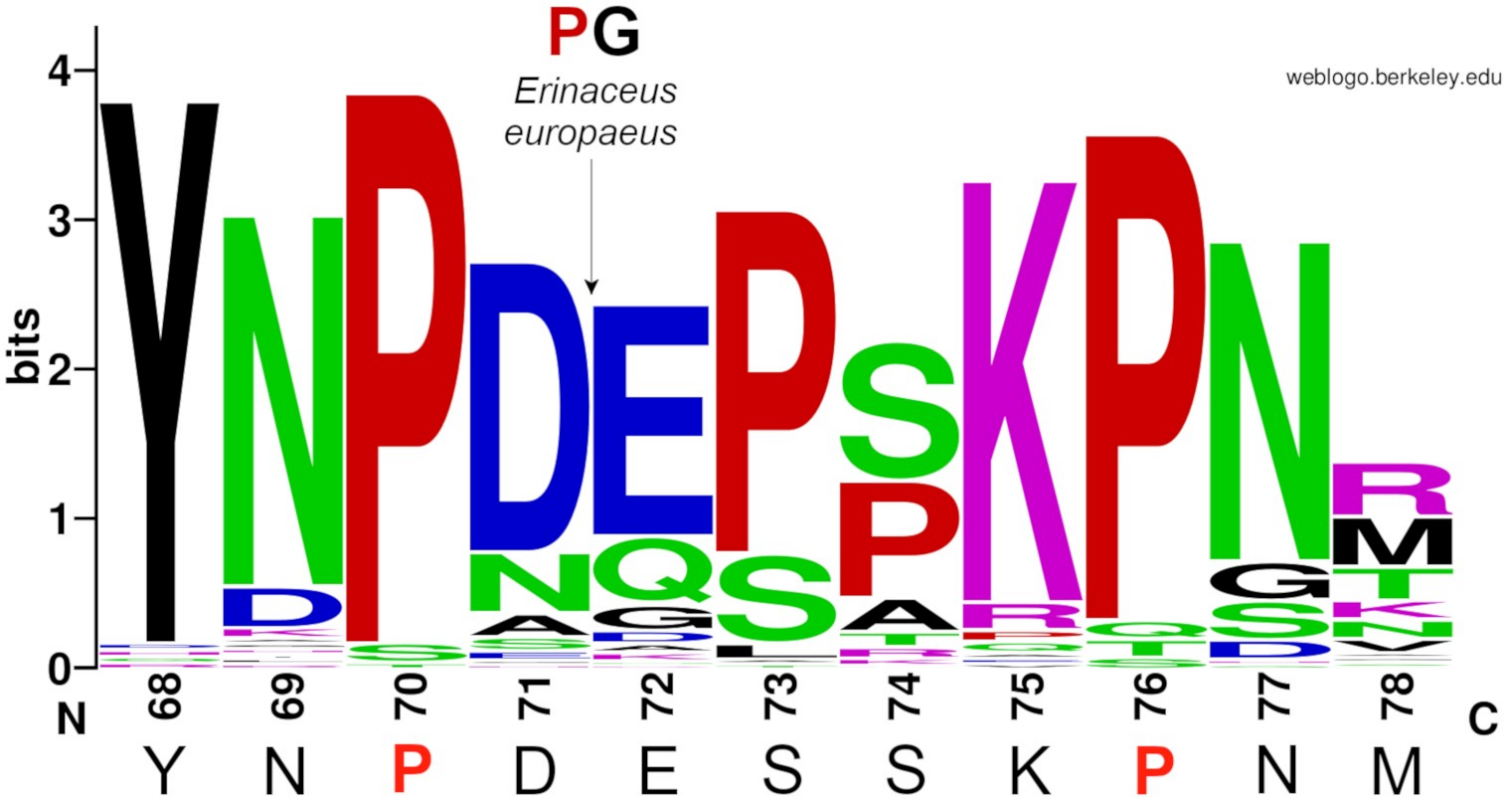
Sequence logo for amino acids γY68–γM78 (numbering according to mature human sequence) presents the conservation of prolines (red) in the unfolded region of the γ chain of fibrinogen. The sequence below the logo belongs to human.

As aforementioned, we have demonstrated that amino acid containing secondary amine on position γP76 is necessary to preserve the C-terminal part of the unfolded γ chain part of coiled-coil connector of fibrinogen. We hypothesize that γP70, or other non-proteinogenic amino acid having secondary amine, is necessary for preserving the N-terminal part of the unfolded region of the α-helix in the γ chain. To support this, a sequence analysis of the unstructured part γ chain of fibrinogen (γY68–γM78 according to human mature γ chain) among 87 mammalian species was performed. Prolines, those appear at six different positions, are highly conserved in this region of fibrinogen (Fig 5). Prolines are found at positions 70 (83 species incl. human), 76 (80 species incl. human), 73 (65 species), 74 (31 species), and 75 (2 species). There are two amino acids inserted between the amino acids 71 and 72 in sequence from European hedgehog (*Erinaceus europaeus*) one of which is proline. Only two species have one proline in this region. Most of the mammalian species (37) have three prolines and others have four (26 species) or two (22 species incl. human) prolines. Such conservation and high abundance of prolines could be of importance. The hypothesis, that concerns the necessity of prolines for preservance of unstructured part of the chain of coiled-coil connector of fibrinogen, is supported by profound knowledge [73] that proline-rich regions occur in protein loops.

The major manifestation of oxidation of γR375 that is located in the γ-nodule of fibrinogen was the release of Ca^2+^ ion from its binding site and disruption of the a-hole. Ca^2+^ is necessary for polymerization of fibrin [74], and hence, its release impairs this process. Release of Ca^2+^ ion was also observed in fibrinogen Osaka V (γR375G; [80]), that indicates the necessity of γR375 for Ca^2+^ binding. Another mutation on the position γR375 is fibrinogen Aguadilla (γR375W; [81]) that is demonstrated by improper folding of fibrinogen resulting into fibrinogen storage disease. Oxidation of γR375 was identified in the fibrinogen treated by NaOCl. On the mesoscopic scale, this reagent also led to the most damaged fibrin network, disruption of polymerization sites, affected ability of fibrin to form fibers and hindered lateral aggregation (thinner fibers) as well as prolongation of strands (bundles). All of this can be considered as another proof of the importance of preserving γR375 for appropriate structure and function of fibrinogen.

The absence of changes in the secondary and tertiary structure caused by the others PTMs does not definitely indicate that these PTMs have no effect on fibrinogen. Because most of them are present at the fibrinogen cleavage sites, either by plasmin or hementin, PTMs of these amino acids may affect fibrinolysis.

## Conclusions

We have demonstrated that oxidation has a serious impact on the structure of fibrin clots and that the extent of changes increases with the oxidative strength of the reagent. Similarly, the PTM count grows with the strength of reagent. Of the 154 PTMs, 19 were induced into the fibrinogen structure by SIN-1, 51 by MDA, and 84 by NaOCl.

We have showed that MD simulations performed with Gromos force field are able to capture the impact of PTMs on fibrinogen structures for isolated fragments of fibrinogen in solution on timescale of 100ns. Interpreting the results, one must be aware of our models are considerably simplified (they consider effect of only a few PTMs on a fragment of fibrinogen) and thus the described structural changes are not necessarily the causes of changes in fibrin nets although they participate in them.

MD simulations revealed that the effect of a given PTM on the fibrinogen structure varies from the negligible alternations to serious disruptions. No considerable changes were observed for the structures γ(Ox)K380, γ(Ox)K381, Bβ(Ox)K133 and γ(Ox)K58. γ(Ox)R375 resulted into the release of Ca^2+^ ion that is preceded by unfolding of the C-terminal α-helix of the P subdomain. This finding can help in explaining the corruption of fibrin network made of fibrin bearing this PTM, as the Ca^2+^ ion is necessary for appropriate fibrin polymerization [74]. Folding of amino acids γE72–γN77 to a short α-helix as a consequence of γ(Ox)P76E, but not of γ(Ox)P76PGA, collectively with conservation of up to four prolines among mammals in the loops γY68–γM78, support the necessity of prolines or other amino acids with secondary amine herein to preserve the disordered nature of this region. We hypothesize that the disordered region of the γ chain (γY68–γM78) destabilizes the coiled-coil structure of fibrinogen in its vicinity by either introducing π-helices (WT, Bβ(Ox)K122, Bβ(Ac)K130, γ(Ox)P76E, γ(Ox)P76PGA, Aα(Ox)M91 and Bβ(Ox)N140) or non-helical secondary structure, usually bends and turns (Bβ(Ac)K122, Bβ(Ac)K133 and Aα(Ox)M91) into the structure of Aα and mainly Bβ chains of fibrinogen. These disturbancies may participate in binding of fibrinolytic enzymes, as binding sites of plasmin and hementin are within the region, where these distrurbancies were observed. MD simulations suggest that the studied PTMs may alter mode of interactions among fibrinogen chains in coiled-coil connector.

The study drew an attention to the issue of natural behaviour and function of the unstructured region of the γ chain of fibrinogen. Such knowledge would help not only to understand an impact of PTMs on fibrinogen structure more in-depth, but especially it would aid in understanding of the internal dynamics of the coiled-coil connector of fibrinogen and its interactions with fibrinolytic molecules those are bound within this region.

With knowledge of the nature and impact of posttranslational modifications, we can better understand the functional and structural properties of fibrinogen. This is the first step in assessing against these pathological states connected with inflammation, thrombotic diseases, atherosclerosis, diabetes, etc. It is of particular clinical significance that these findings suggest specific disparate therapies that will be most effective at different stages of fibrin participation in thrombus development.

This study is the first step in our current effort to characterize the PTMs typical for cardiovascular diseases and describe their impact on fibrinogen structure and function.

## Supporting information

S1 Appendix Detailed description of MD simulations protocol. (PDF)

S1 Table List of all PTMs detected in fibrinogen by MS. Amino acid numbering according to mature protein chains. Abbreviations used (alphabetically): ace = acetaldehydation, ami = amination, chl = chloration, Dha = docosahexaenoic acid, dic = dichloration, dio = dioxidation, HNE = hydroxynonenal, hky = hydroxykynurenine, kyn = kynurenine, mal = malonylation, met = methylation, nit = nitration, oxa = oxalacetate, oxi = oxidation, pyE = pyroglutamate.
(PDF)

S1 Fig Simulated parts of fibrinogen. Crystal structure 3GHG with highlighted regions used for MD simulations. γ-nodule contains amino acids γ148–γ394, coiled-coil connector contains amino acids Aα70–Aα126, Bβ101–Bβ157, and γ47–γ97. Aα chain is presented in magenta, Bβ chain in red, and γ chain in orange. Note that following parts of fibrinogen are missing in the crystal structure: Aα1–Aα26, Aα213–Aα610, Bβ1–Bβ57, Bβ459–Bβ461, γ1, γ396–γ411.
(PDF)

S2 Fig Chemical formulae of modified amino acids. Chemical formulae of non-proteinogenic amino acids those were introduced into fibrinogen structure as a result of PTM. Formulae of the original proteinogenic amino acids are shown as well.
(PDF)

S3 Fig Development of RMSD of C_α_ carbons in time for coiled-coil connector systems. (PDF)

S4 Fig Development of radius of gyration of C_α_ carbons in time for coiled-coil connector systems. (PDF)

S5 Fig RMSF of C_α_ carbons computed over the last 25 ns of simulations of the coiled-coil connector systems. RMSF for each fibrinogen chain is shown separately.
(PDF)

S6 Fig Development of secondary structure (DSSP) in time for coiled-coil connector systems. Positions of the modified amino acids are highlighted by red bars at sides of plot.
(PDF)

S7 Fig Characterization of kinks induced into the α-helices by their partial switch to π-helices. The decrease of the angle in Bβ(Ox)K122 and Aα(Ox)M91 may be caused by refolding of the α-helix.
(PDF)

S8 Fig Development of RMSD of C_α_ carbons in time for γ-nodule systems. (PDF)

S9 Fig Development of radius of gyration of C_α_ carbons in time for γ-nodule systems. (PDF)

S10 Fig Development of secondary structure (DSSP) in time for systems examining γ-nodule of fibrinogen. Positions of the modified amino acids are highlighted by red bars at sides of plots.
(PDF)

S11 Fig Unbinding of the Ca^2+^ ion. Distance of Ca^2+^ ion from C_α_ carbons of γD318 (black), resp. γD320 (red) and from C carbon of γF322 (green) in dependence of time.
(PDF)

S12 Fig Hydrogen bond network formed as a result of γ(Ox)R375. Green lines show hydrogen bonds formed in the C-terminal part of the system with oxidized γR375. In the WT simulation γR375 forms hydrogen bond only with γK373 (shown in violet). Carbon is shown in cyan, hydrogen in white, oxygen in red, nitrogen in blue and Ca^2+^ ion in black.
(PDF)

S13 Fig RMSF of C_α_ carbons computed over the last 50 ns of simulations of the γ-nodule systems. (PDF)

S14 Fig Ramachandran plot for selected amino acids of the γ chain. Ramachandran plot for amino acids γ50–γ90 of fibrinogen depicts γM78 in the region typical for collagen triple helix. The other amino acids nearby belong to the unfolded region of the γ chain. Plot was made by Procheck.
(PDF)
