# Supplementary material for: Impact of posttranslational modifications on atomistic structure of fibrinogen
Source: PLoS One. 2020 Jan 29;15(1):e0227543. doi: 10.1371/journal.pone.0227543 (PMC6988951; doi:10.1371/journal.pone.0227543)
Supplement: S1 Appendix — (PDF) [file pone.0227543.s001.pdf]

Appendix S1: Detailed description of MD simulation settings

Initial protein coordinates for MD simulations were obtained from crystal structure 3GHG [1], that was shortened in SwissPDBViewer [2]. To describe an effect of PTMs on γ-nodule, amino acids 148–394 of the C chain and Ca^{2+} ion were used. For modeling the coiled-coil connector of fibrinogen, amino acids 70–126 of the A chain of 3GHG, 101–157 of the B chain and 47–97 of the C chain were used. Selected PTM were introduced into the fibrinogen structure by either Vienna-PTM 2.0 [3]. Each PTM was treated in a separate simulation and its behavior was compared with simulation of WT system.
MD simulations were performed in Gromacs 5.1.1 [4] with Gromos 54a7 [5] force field that was recently extended for parameters for post-translationally modified amino acids [6]. Proteins were solvated by SPC water and, when needed, counterions were added to neutralize the cubic simulation box whose dimension is by 1 nm larger than the longest dimension of the protein. Systems underwent 1000 steps of steepest descent geometry optimization and accordingly were equilibrated by 25 ps MD simulation with timestep of 1 fs. This simulation uses the same setting as production simulation. Production simulations with the time-step of 2 fs were performed for 100 ns with the last 25 ns used for analyses of coiled-coil connector and the last 50 ns of 250 ns simulation was used for analyses simulations of γ-nodule. Simulation were performed at physiological temperature 310 K maintained by Bussi's velocity rescaling thermostat [7] with coupling constant of 1 ps, that was simultaneous applied to protein (incl. Ca^{2+} for simulations of γ-nodule) and solution (SPC water [8] and eventually counterions). Pressure was kept isotropically at 1 atm by Berendsen barostat [9] with coupling constant of 5.1 ps and compressibility of 4.5×10^{-5} bar^{-1}. Long range interactions were treated in the pairwise manner up to 1.4 nm from a particle. Beyond, Lennard-Jones interactions were cut and electrostatic interactions were approximated by reaction field [10] approach with relative dielectric constant of 65. Simulations of the γ-nodule were not able to preserve Ca^{2+} at its position as determined from the crystal structure with reaction-field electrostatics. Alternation of treatment of long range coulombic interaction to particle-mesh Ewalds, PME [11], solved this issue. LINCS [12] were used for all bonds. Periodic boundary conditions were used. Geometry of resultant structures was once more optimized my steepest descent technique.
Frames taken every 250 ps were used for analyses, those were performed by standard tools of Gromacs. Secondary structure was described by DSSP [13] as implemented in Gromacs (`gmx do_dssp`). Existence of hydrogen bond was defined by its presence in at least in 50% of frames in the last 40 ns (coiled-coil systems) resp. 50 ns (γ-nodule domain systems) of simulations. Root mean square fluctuation computed by `gmx rmsf` command of Gromacs, root mean square deviation (`gmx rms`) and radius of gyration (`gmx gyrate`) were computed only for C_α carbons of each amino acid. Kink within the coiled-coil connector were computed by `gmx bundle` command and used three N- resp. C-terminal C_α carbons those preserved α-helical structure (according to DSSP analysis) to define the axis of helix and C_α carbons of the p-helix (according to DSSP analysis) to define the kink.
Ramachandran plot was computed by Procheck [14] from the last frame of simulation, whose geometry was optimized by steepest descent algorithm as implemented in Gromacs.
Sequence analysis was performed on 87 mammalian protein sequences of the γY68–γM78 region (notion according to human mature γ chain of fibrinogen) of the fibrinogen γ chain, those were downloaded from the NIH database [15] using keyword FGG. Only one splicing variant was used for each species (both splicing variants of the γ chain of fibrinogen are identical in the region of interest). Sequences were aligned by ClustalX [16]. Sequence logo was created by WebLogo web server [17] and adjusted manualy (insertion in sequence of European hedgehog, *Erinaceus europaeus*) in Inkscape.
