# Supplementary material for: Impact of posttranslational modifications on atomistic structure of fibrinogen
Source: PLoS One. 2020 Jan 29;15(1):e0227543. doi: 10.1371/journal.pone.0227543 (PMC6988951; doi:10.1371/journal.pone.0227543)
Supplement: S1 Table — Amino acid numbering according to mature protein chains. Abbreviations used (alphabetically): ace = acetaldehydation, ami = amination, chl = chloration, Dha = docosahexaenoic acid, dic = dichloration, dio = dioxidation, HNE = hydroxynonenal, hky = hydroxykynurenine, kyn = kynurenine, mal = malonylation, met = methylation, nit = nitration, oxa = oxalacetate, oxi = oxidation, pyE = pyroglutamate. (PDF) [file pone.0227543.s002.pdf]

PTMs detected in fibrinogen by mass spectrometry

**Aα chain**				
AA	*pos*	*NaOCl*	*MDA*	*SIN-1*
D	32	oxi		
P	34	oxi		
Y	43	ami		
D	56	met		
K	70	oxi	oxi	
K	123		HNE	
N	139	oxi		
R	141	dio		
R	141	oxi		
K	148	dio		
K	176	oxi		
E	179	met		met
P	209		oxi	
D	212	oxi		
D	234	oxi		
N	269	oxi		
P	270	dio		
W	276	kyn		
W	276	dio		dio
W	276	hky		
N	277			met
D	477	oxi		
D	477	met		
K	508		oxi	
F	513	dio		
F	514		dio	
P	516	pyE		
P	516	dio		
E	520		met	
P	546	oxi	pyE	
E	578	met		
D	586	oxi		
K	601		HNE	

**Bβ chain**				
AA	*pos*	*NaOCl*	*MDA*	*SIN-1*
E	25	met	met	
P	27	pyE	pyE	
F	115	oxi		oxi
Y	117	oxi		
K	122	oxi	ace	oxi
K	130		ace	
K	133	oxi	ace	
D	134	met	met	
E	141		met	
Y	142	ami		
E	147		met	
H	149	oxi	oxi	
Y	152		oxi	
Y	152	ami	ami	
D	154		oxi	
D	154	met	met	
S	184		mal	
K	217	oxi		oxi
E	220		met	
Y	225	ami		ami
Y	239		ami	
Y	239		chl	dic
D	240			met
E	245		met	
N	246	oxi		
W	249	dio	oxa	
W	249		hky	
W	249		kyn	
F	273	oxi	oxi	
Y	285	ami		
Y	285	dic		
P	289			oxi
Y	292	ami	dio	
W	293	oxi	dio	oxa
P	307	dio		
W	317	oxi	dio	
W	317		kyn	
W	317		oxa	
K	318		oxi	
K	318		dio	
H	325	oxi	oxi	
Y	326		ami	
E	334	met	met	
H	370	oxi		
N	371	oxi		
D	381	oxi		
D	383	oxi		

**γ chain**				
AA	*pos*	*NaOCl*	*MDA*	*SIN-1*
E	13			met
K	58	oxi		
P	70	pyE		
P	70	dio		
E	72	met		
P	76	pyE	pyE	
D	80	met		
Y	96	ami	nit	
Y	96	chl		
E	97	met	met	
Y	109	dic		ami
K	120	oxi		
E	213	met	met	
F	215	dio		
H	217		oxi	
P	220		oxi	
P	220		pyE	
F	226	dio		
W	227	oxa		oxa
K	232	oxi		
F	322			dio
H	340	oxi		
H	343	oxi		
N	345	oxi	oxi	
R	375	oxi		
K	380	oxi		
K	381	oxi		
K	406	oxi		

D	383	met		
W	385	oxa		
W	385	dio		
D	389		met	
C	394	mal		
S	395	mal		
W	419		oxi	
Y	422			oxi
W	437	oxi	oxi	
K	441	oxi		oxi
K	453			oxi
F	458			oxi

Table S1. List of all PTMs detected in fibrinogen by MS. Amino acid numbering according to mature protein chains. Abbreviations used (alphabetically): ace = acetaldehydation, ami = amination, chl = chloration, Dha = docosahexaenoic acid, dic = dichloration, dio = dioxidation, HNE = hydroxynonenal, hky = hydroxykynurenine, kyn = kynurenine, mal = malonylation, met = methylation, nit = nitration, oxa = oxalacetate, oxi = oxidation, pyE = pyroglutamate
