# Supplementary material for: Impact of posttranslational modifications on atomistic structure of fibrinogen
Source: PLoS One. 2020 Jan 29;15(1):e0227543. doi: 10.1371/journal.pone.0227543 (PMC6988951; doi:10.1371/journal.pone.0227543)
Supplement: S1 Fig — Crystal structure 3GHG with highlighted regions used for MD simulations. γ-nodule contains amino acids γ148–γ394, coiled-coil connector contains amino acids Aα70–Aα126, Bβ101–Bβ157, and γ47–γ97. Aα chain is presented in magenta, Bβ chain in red, and γ chain in orange. Note that following parts of fibrinogen are missing in the crystal structure: Aα1–Aα26, Aα213–Aα610, Bβ1–Bβ57, Bβ459–Bβ461, γ1, γ396–γ411. (PDF) [file pone.0227543.s003.pdf]

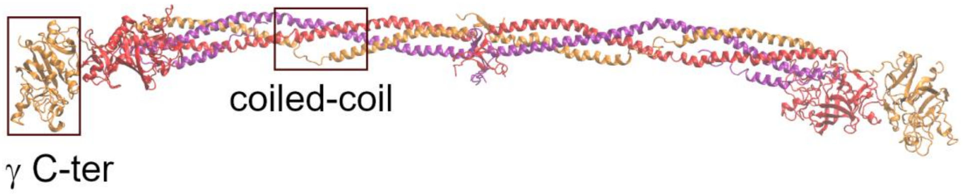

Fig S1. Simulated parts of fibrinogen. Crystal structure 3GHG with highlighted regions used for MD simulations. γ-nodule contains amino acids γ148 – γ394, coiled-coil connector contains amino acids Aα70 – Aα126, Bβ101 – Bβ157 and γ47 – γ97. Aα chain is shown in magenta, Bβ chain in red and γ chain in orange. Note that following parts of fibrinogen are missing in the crystal structure: Aα1 – Aα26, Aα213 – Aα610, Bβ1 – Bβ57, Bβ459 – Bβ461, γ1, γ396 – γ411.
