# Supplementary material for: Impact of posttranslational modifications on atomistic structure of fibrinogen
Source: PLoS One. 2020 Jan 29;15(1):e0227543. doi: 10.1371/journal.pone.0227543 (PMC6988951; doi:10.1371/journal.pone.0227543)
Supplement: S2 Fig — Chemical formulae of non-proteinogenic amino acids those were introduced into fibrinogen structure as a result of PTM. Formulae of the original proteinogenic amino acids are shown as well. (PDF) [file pone.0227543.s004.pdf]

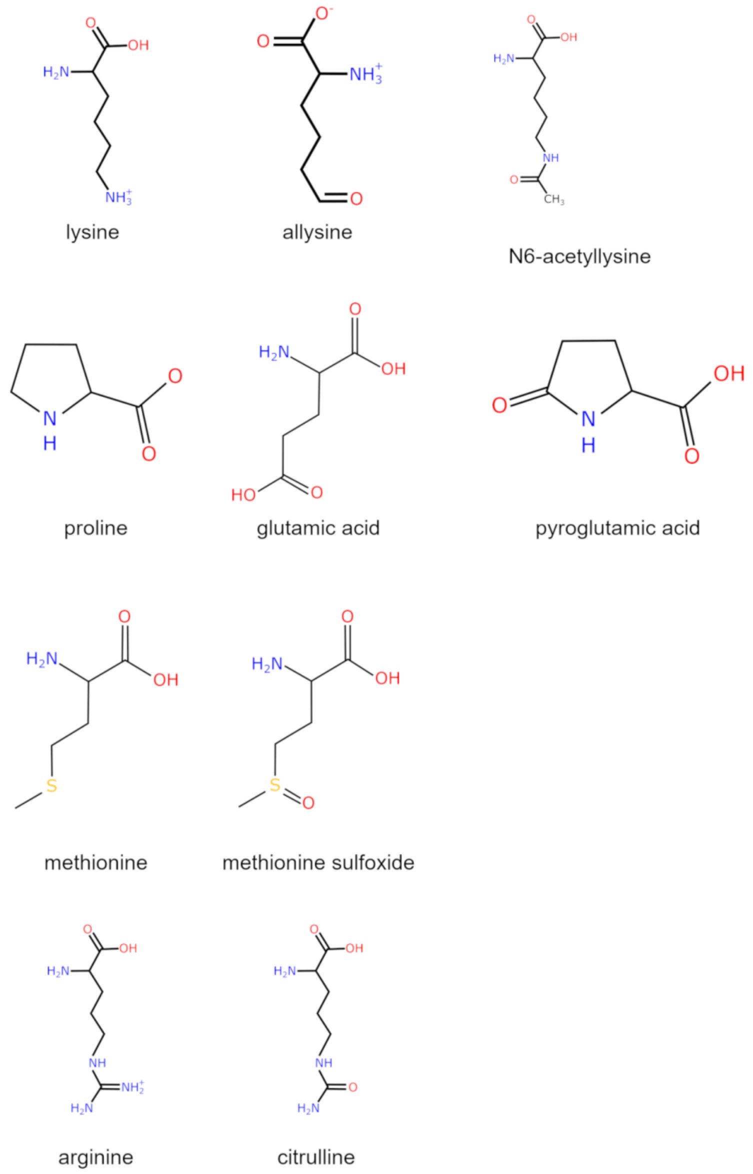

Fig S2. Chemical formulae of non-proteinogenic amino acids those were introduced into fibrinogen structure as a result of PTM. Formulae of the original proteinogenic amino acids are shown as we
