# Supplementary figures and images for: Impact of posttranslational modifications on atomistic structure of fibrinogen
Source: PLoS One. 2020 Jan 29;15(1):e0227543. doi: 10.1371/journal.pone.0227543 (PMC6988951; doi:10.1371/journal.pone.0227543)

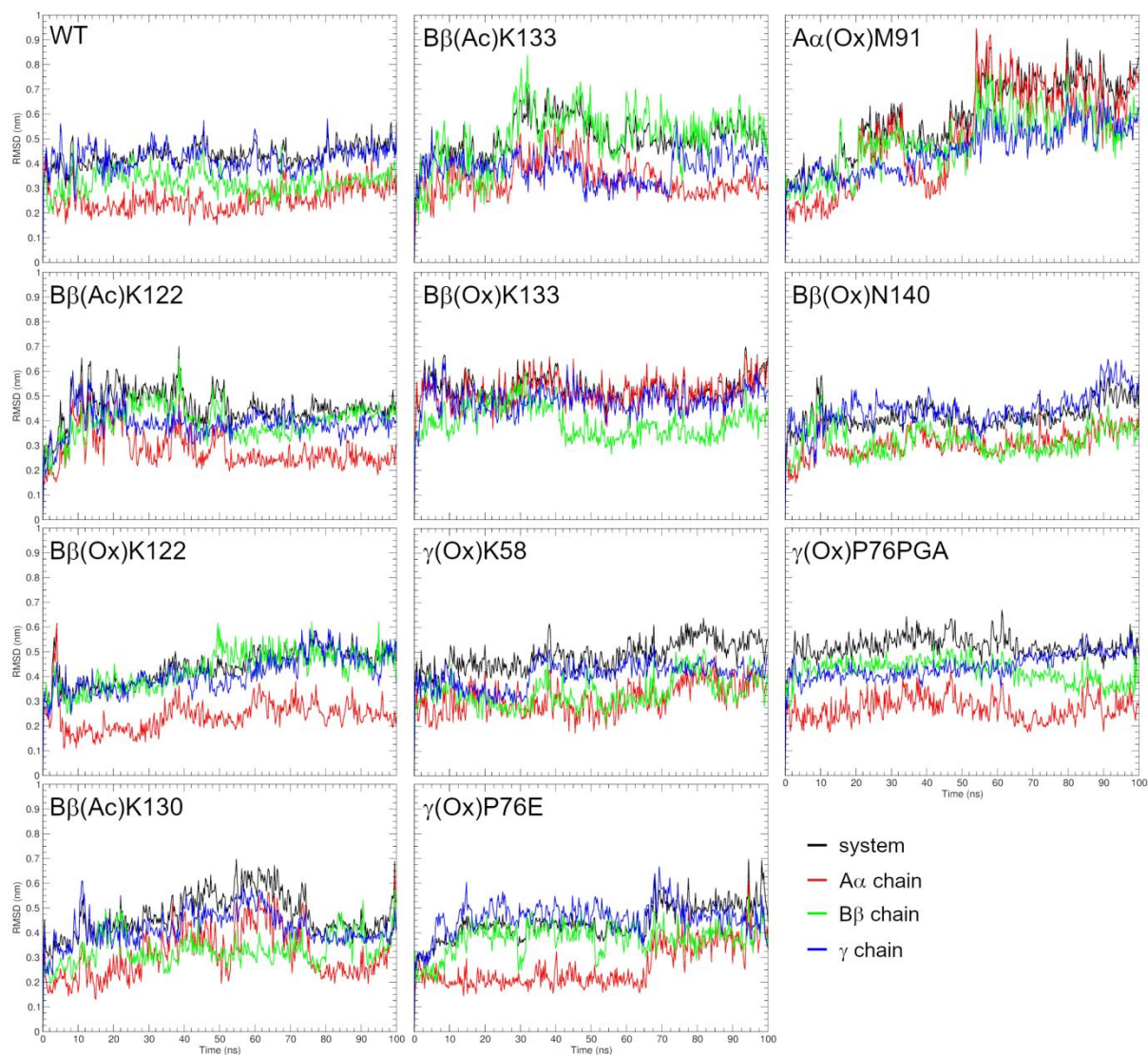

**Fig S3.** Development of RMSD of C $\alpha$  carbons in time for coiled-coil connector systems.

Supplement: S3 Fig — (PDF) [file pone.0227543.s005.pdf]

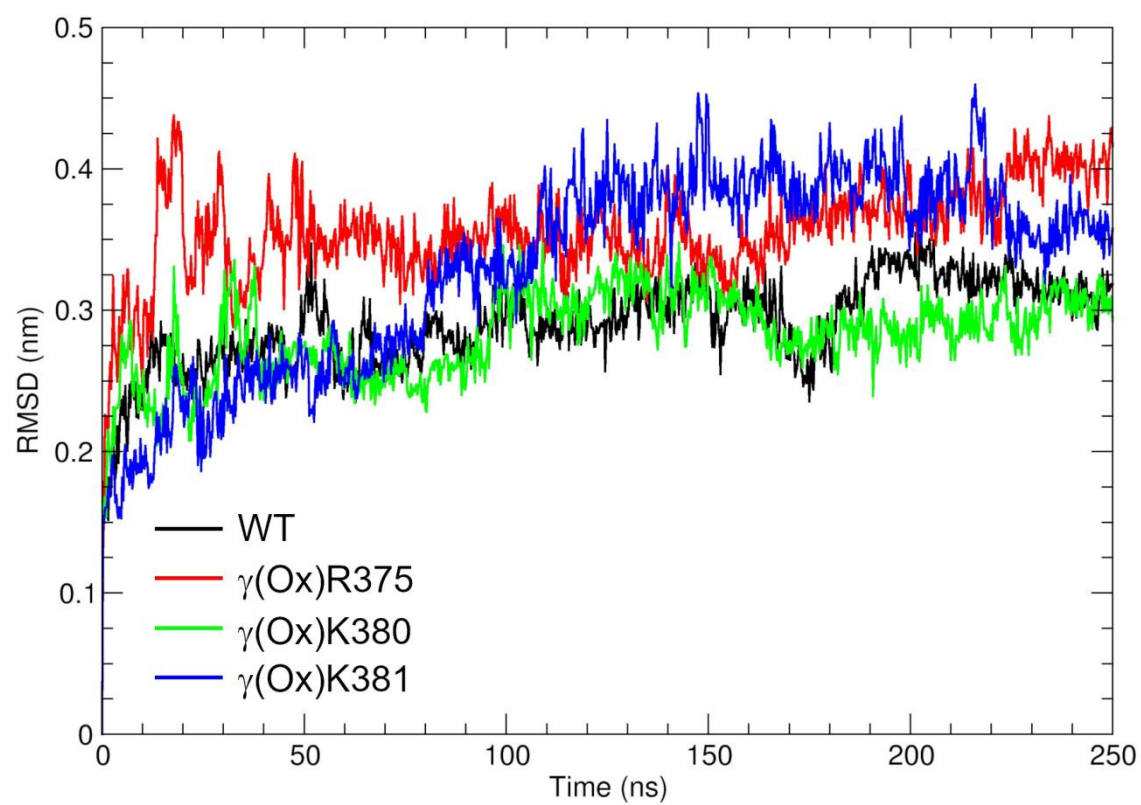

**Fig S8.** Development of RMSD of C $\alpha$  carbons in time for  $\gamma$ -nodule systems.

Supplement: S8 Fig — (PDF) [file pone.0227543.s010.pdf]

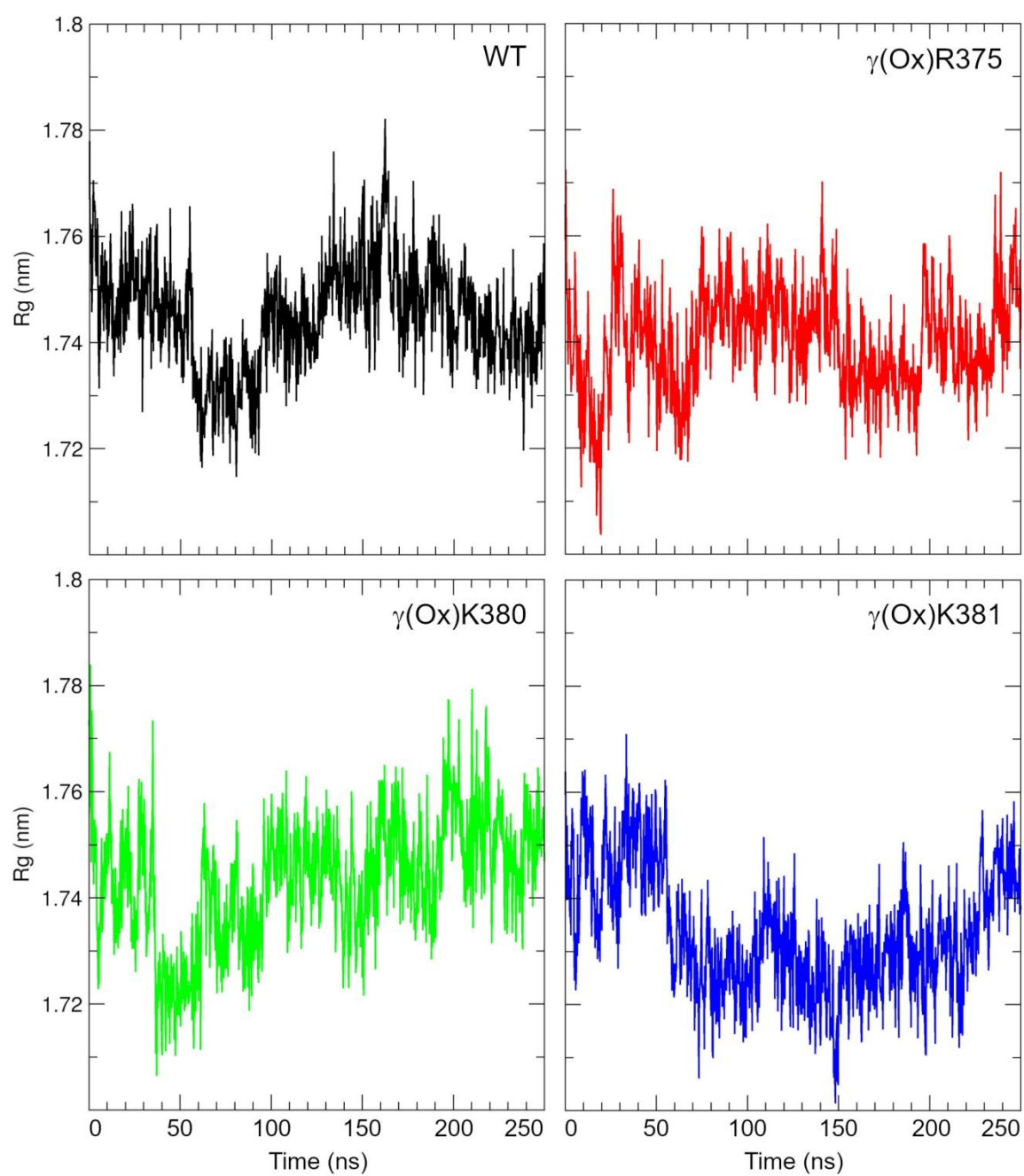

**Fig S9.** Development of radius of gyration of  $C_\alpha$  carbons in time for  $\gamma$ -nodule systems.

Supplement: S9 Fig — (PDF) [file pone.0227543.s011.pdf]
