# Supplementary material for: Impact of posttranslational modifications on atomistic structure of fibrinogen
Source: PLoS One. 2020 Jan 29;15(1):e0227543. doi: 10.1371/journal.pone.0227543 (PMC6988951; doi:10.1371/journal.pone.0227543)
Supplement: S4 Fig — (PDF) [file pone.0227543.s006.pdf]

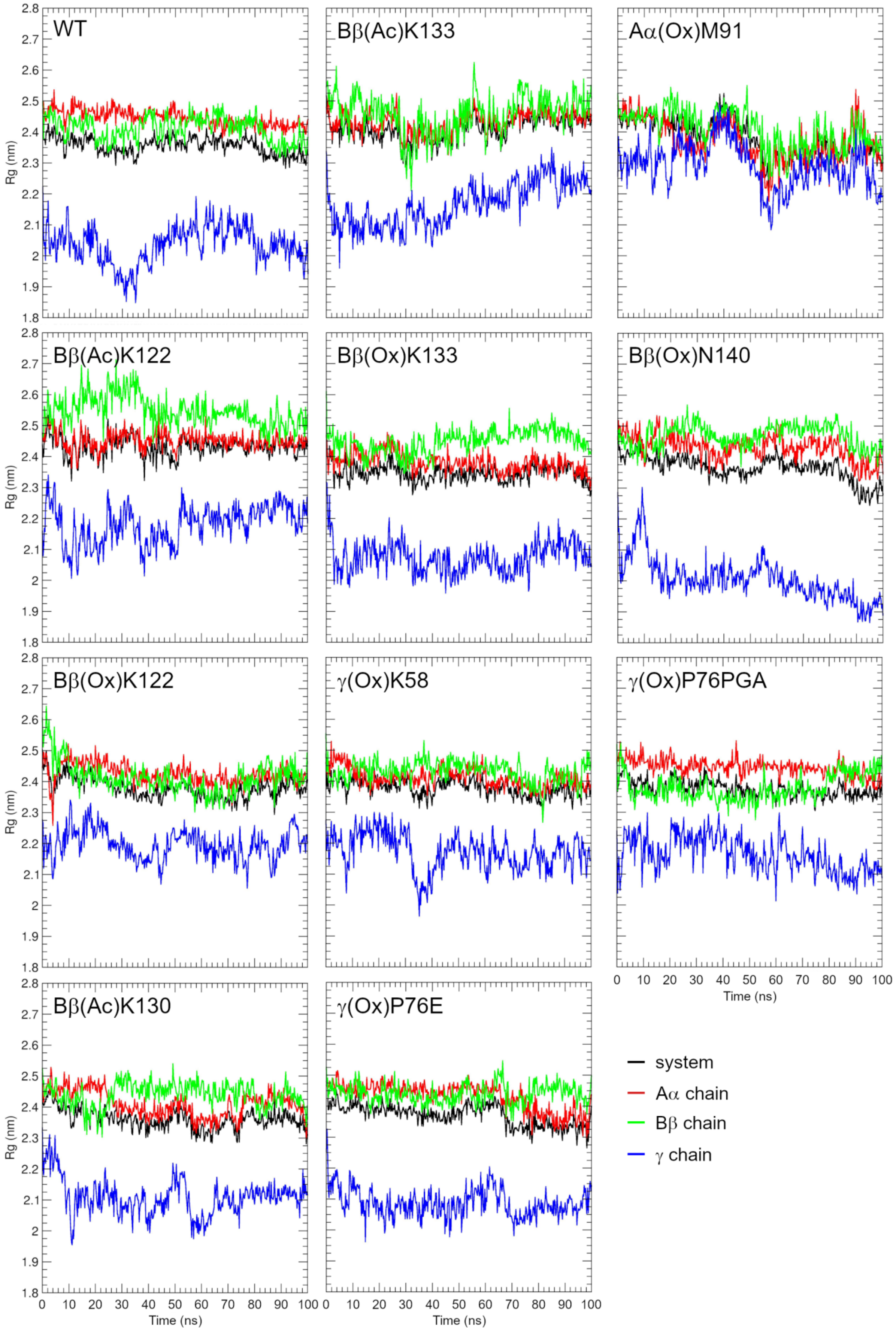

Fig S4. Development of radius of gyration of C_α carbons in time for coiled-coil connector systems.
