# Supplementary material for: Impact of posttranslational modifications on atomistic structure of fibrinogen
Source: PLoS One. 2020 Jan 29;15(1):e0227543. doi: 10.1371/journal.pone.0227543 (PMC6988951; doi:10.1371/journal.pone.0227543)
Supplement: S5 Fig — RMSF for each fibrinogen chain is shown separately. (PDF) [file pone.0227543.s007.pdf]

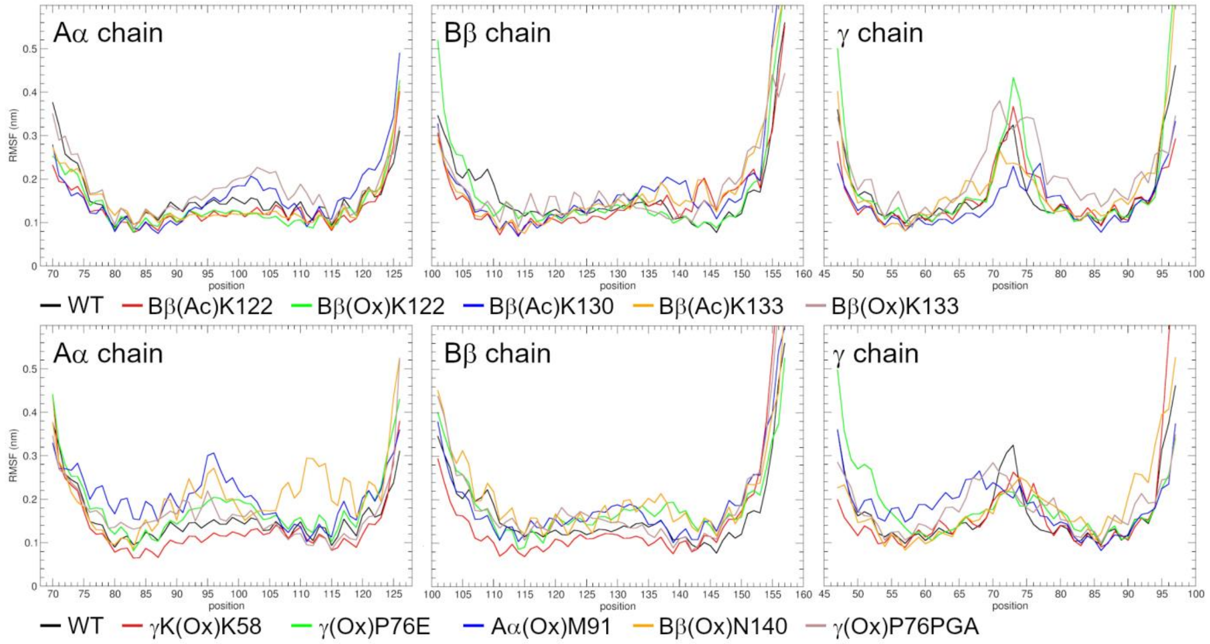

Fig S5. RMSF of C_α carbons computed over the last 25 ns of simulations of the coiled-coil connector systems. RMSF for each fibrinogen chain is shown separately.
