# Supplementary material for: Impact of posttranslational modifications on atomistic structure of fibrinogen
Source: PLoS One. 2020 Jan 29;15(1):e0227543. doi: 10.1371/journal.pone.0227543 (PMC6988951; doi:10.1371/journal.pone.0227543)
Supplement: S6 Fig — Positions of the modified amino acids are highlighted by red bars at sides of plot. (PDF) [file pone.0227543.s008.pdf]

WT

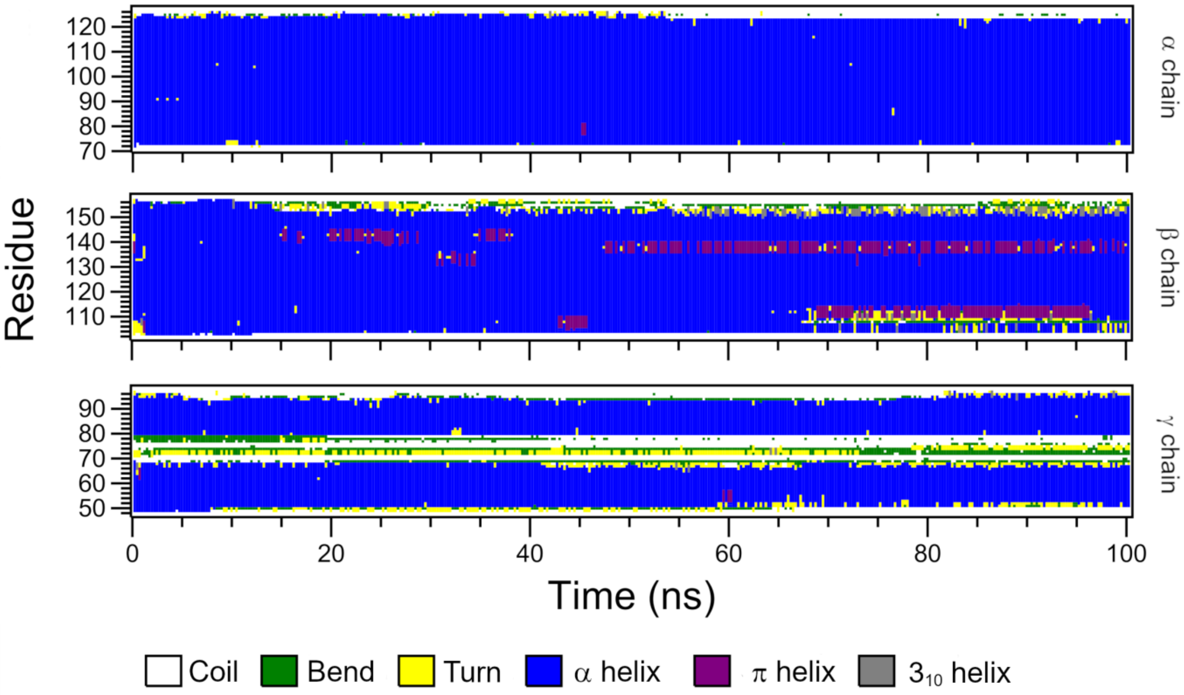
Residue
120
110
100
90
80
70
α chain
150
140
130
120
110
β chain
90
80
70
60
50
γ chain
0
20
40
60
80
100
Time (ns)
Coil
Bend
Turn
α helix
π helix
3₁₀ helix

Bβ(Ac)K122

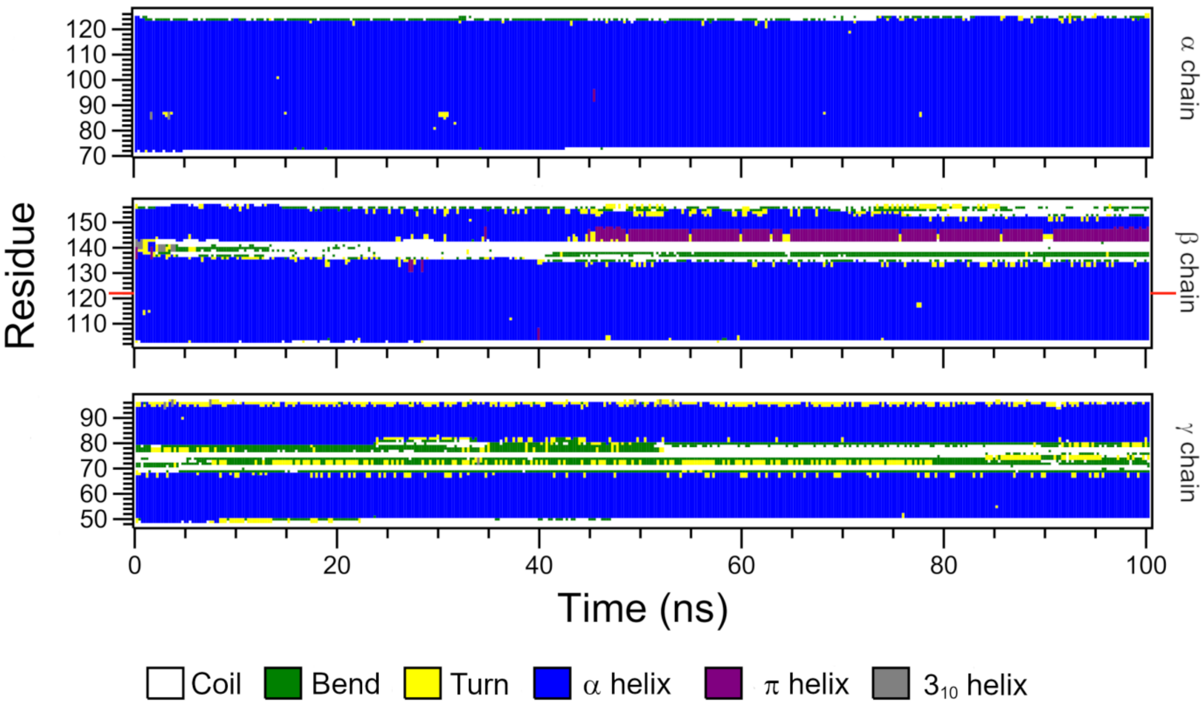
Residue
120
110
100
90
80
70
α chain
150
140
130
120
110
β chain
90
80
70
60
50
γ chain
0
20
40
60
80
100
Time (ns)
Coil
Bend
Turn
α helix
π helix
3₁₀ helix

Bβ(Ox)K122

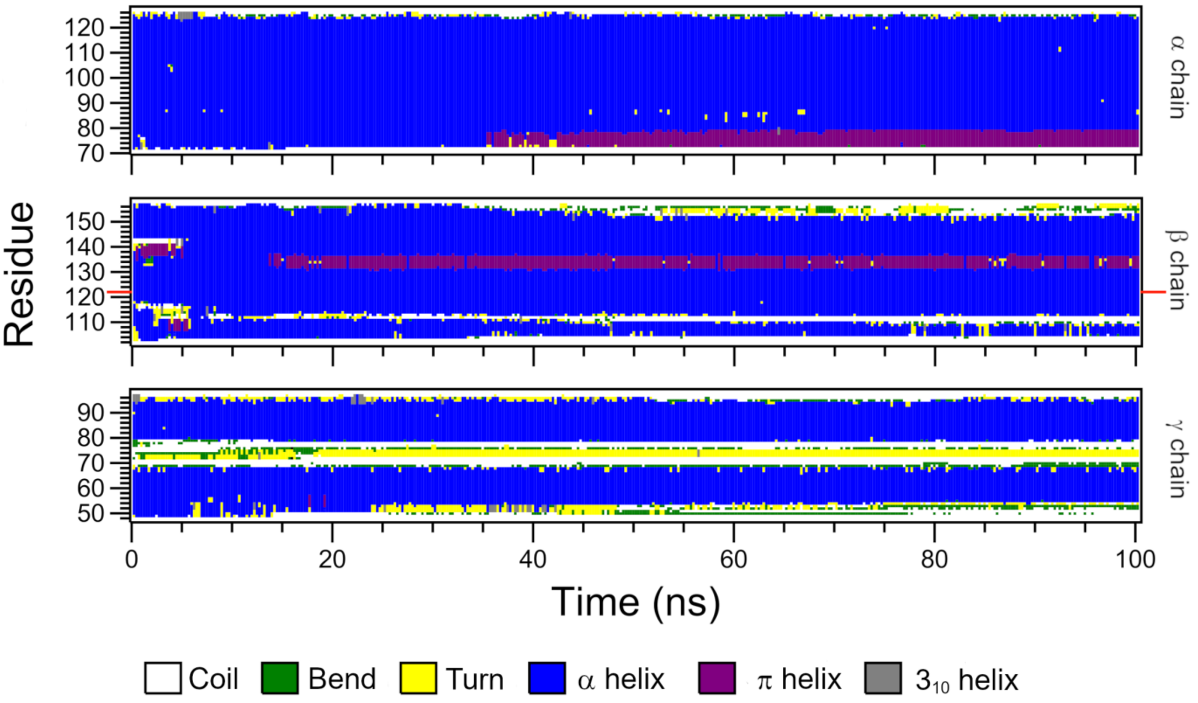

Bβ(Ac)K130

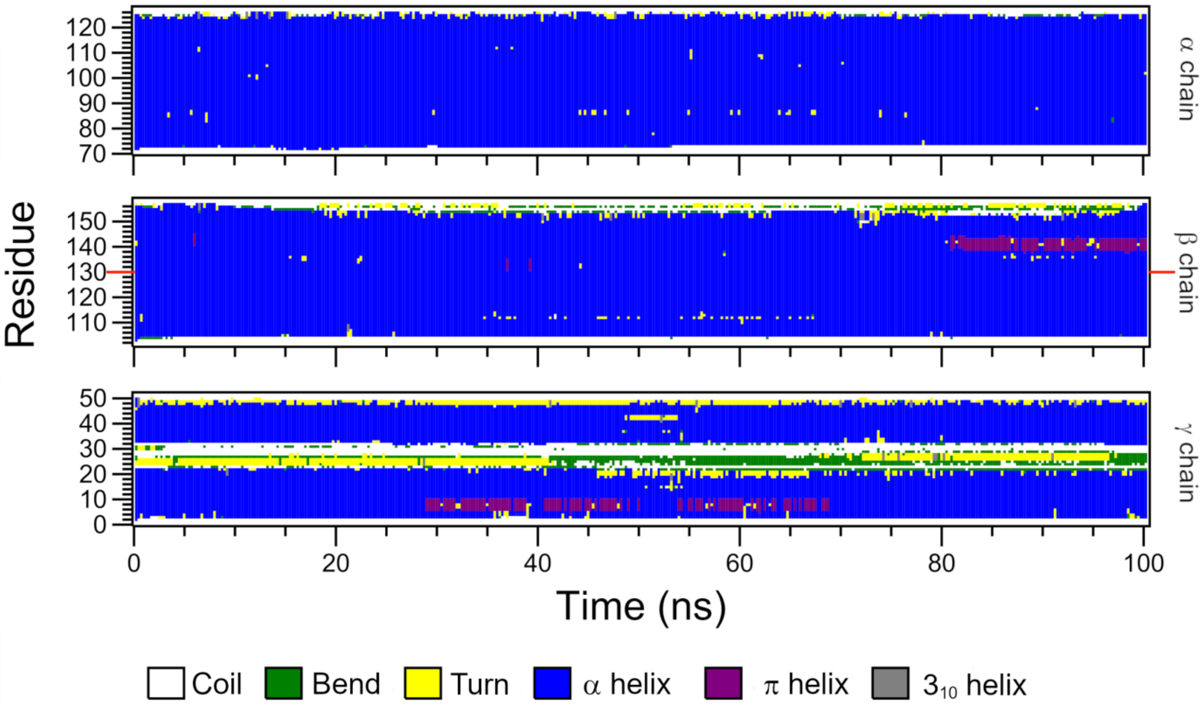

Bβ(Ac)K133

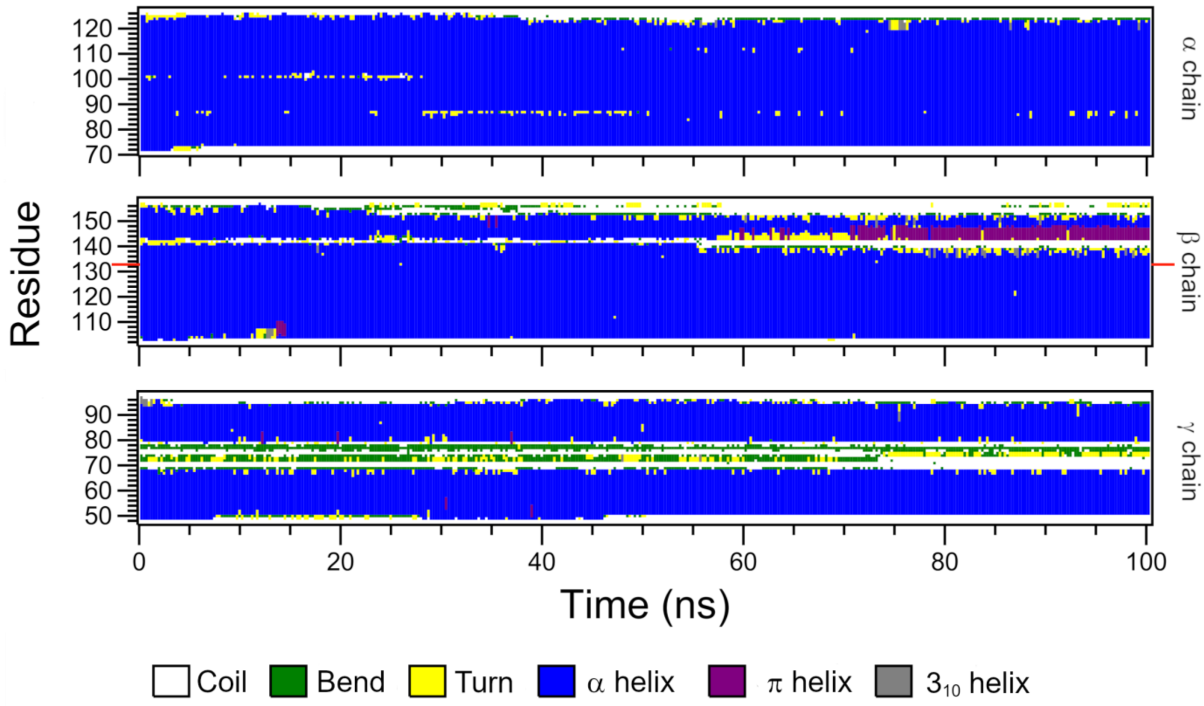

Bβ(Ox)K133

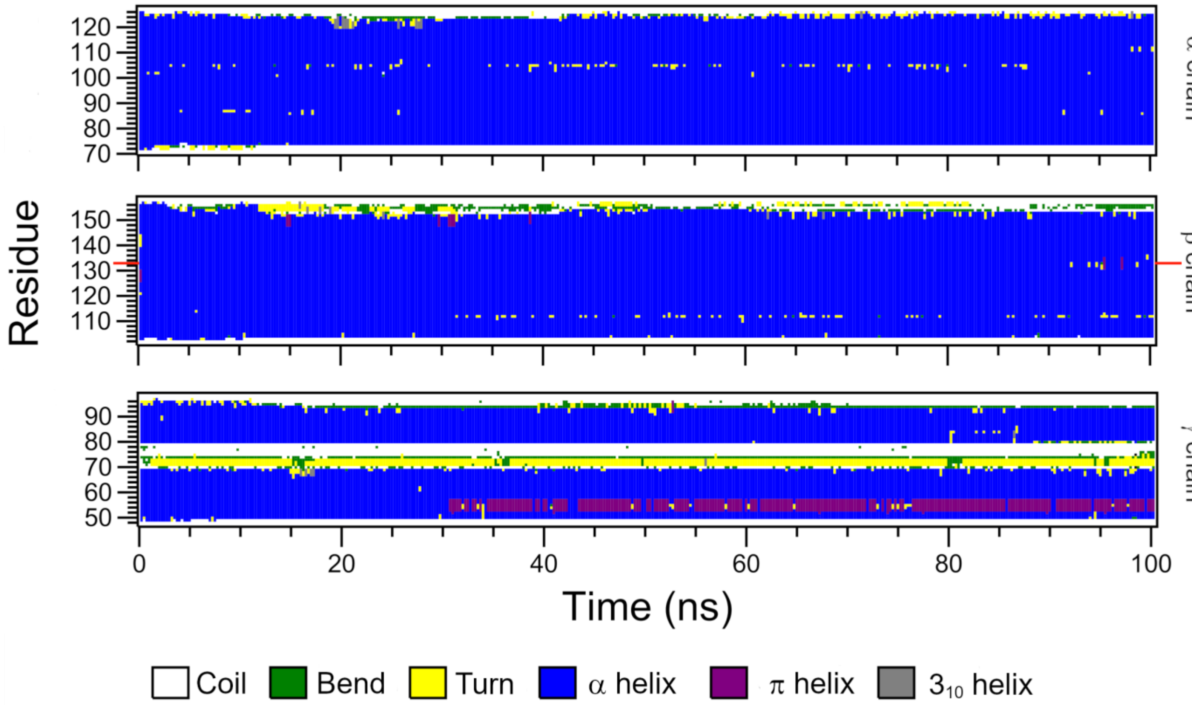

γ(Ox)K58

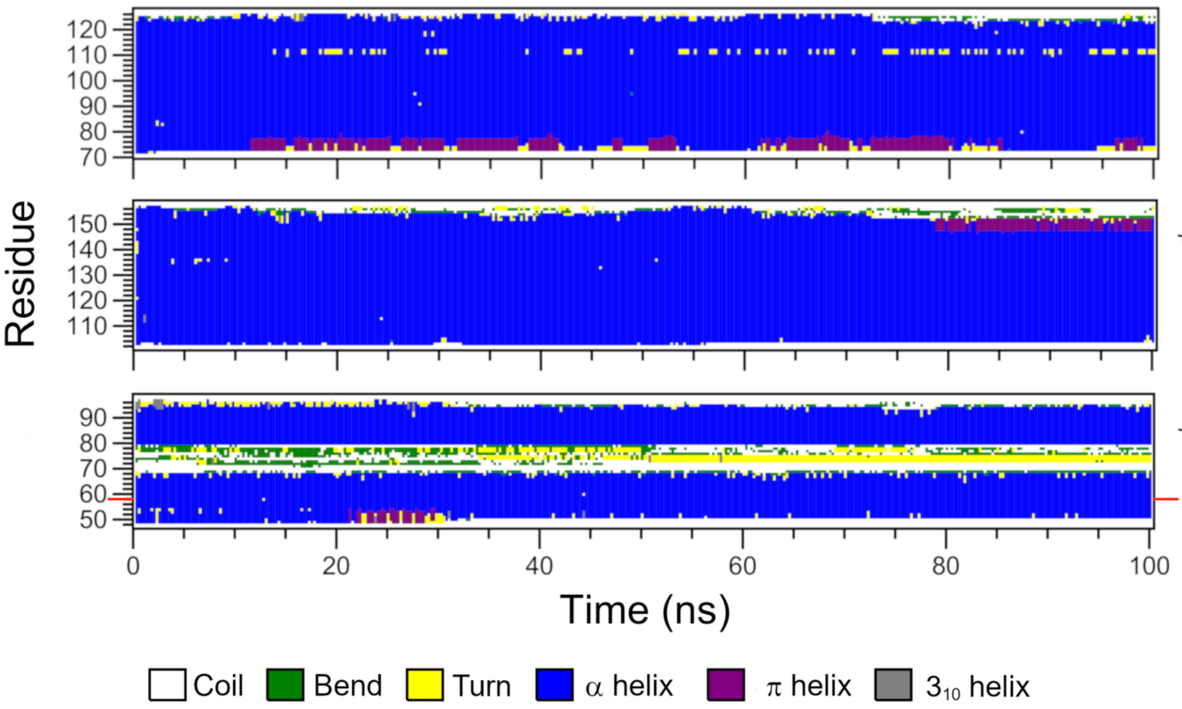

γ(Ox)P76E

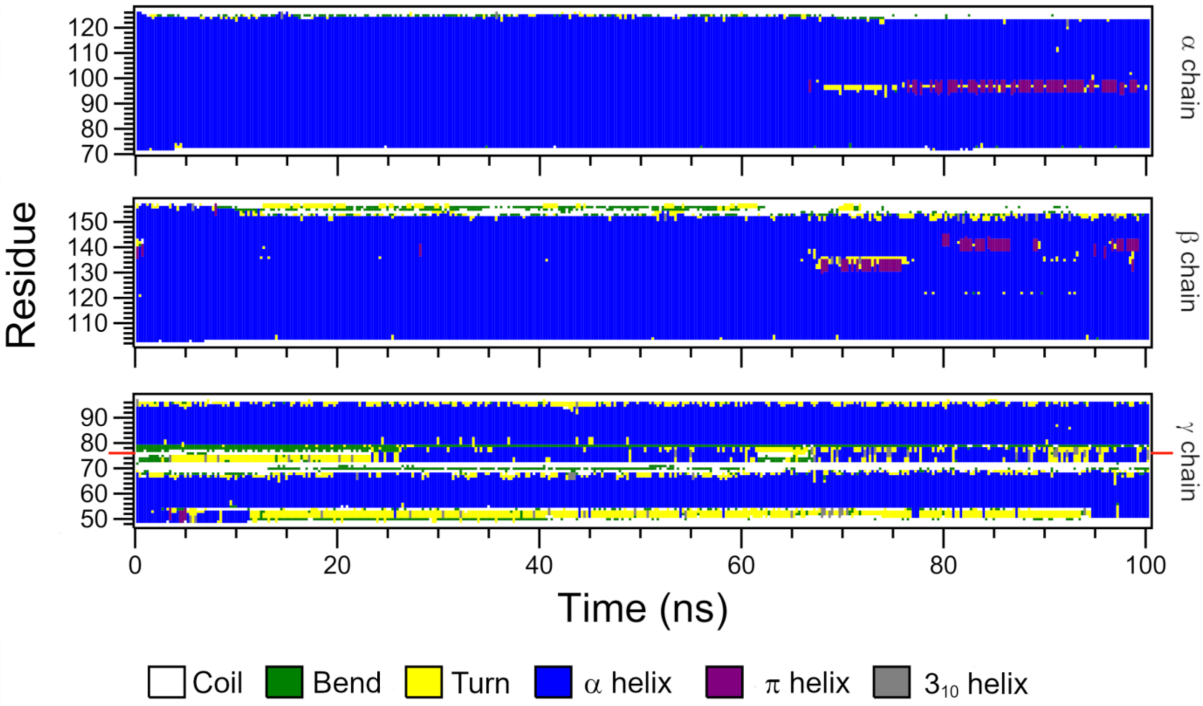

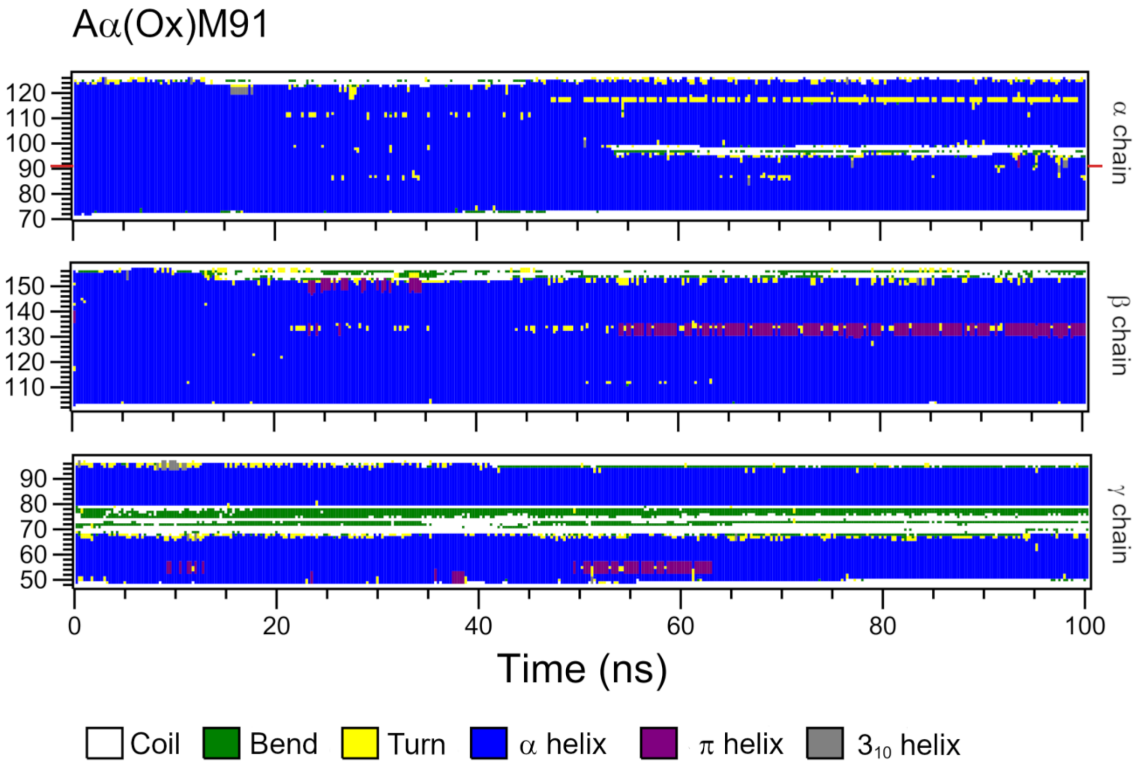

Aα(Ox)M91
120
110
100
90
80
70
α chain
150
140
130
120
110
β chain
90
80
70
60
50
γ chain
0
20
40
60
80
100
Time (ns)
Coil
Bend
Turn
α helix
π helix
3₁₀ helix

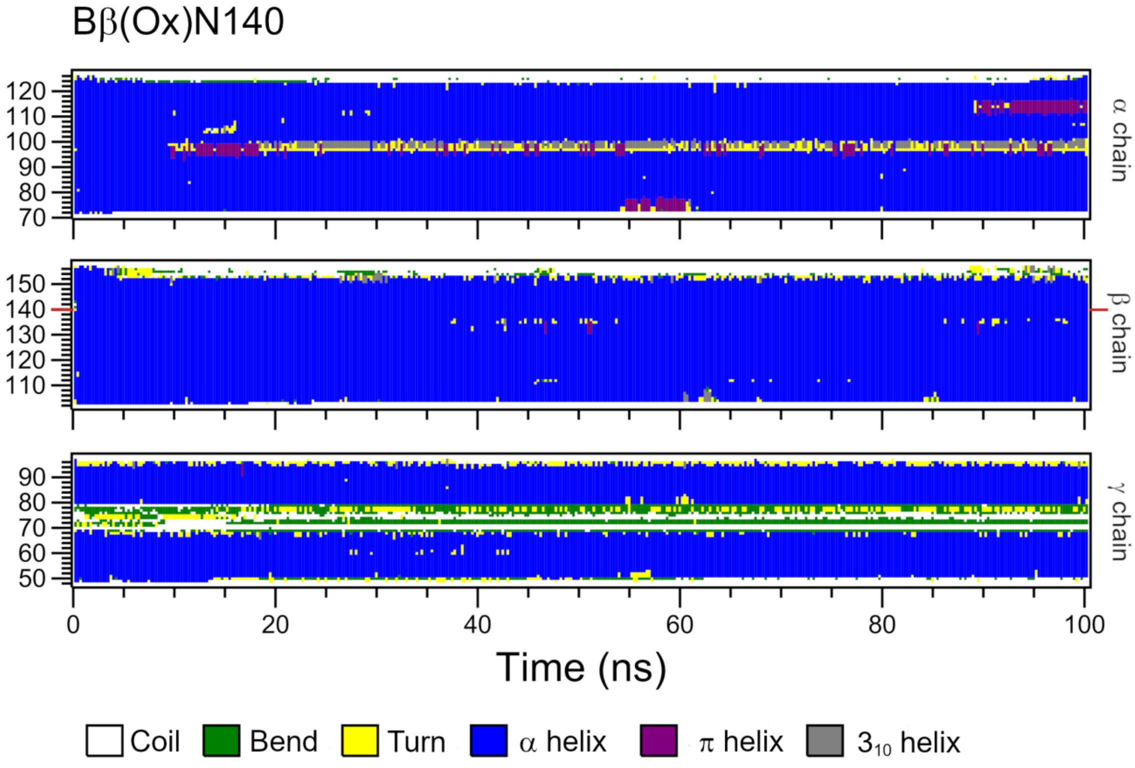

Bβ(Ox)N140
120
110
100
90
80
70
α chain
150
140
130
120
110
β chain
90
80
70
60
50
γ chain
0
20
40
60
80
100
Time (ns)
Coil
Bend
Turn
α helix
π helix
3₁₀ helix

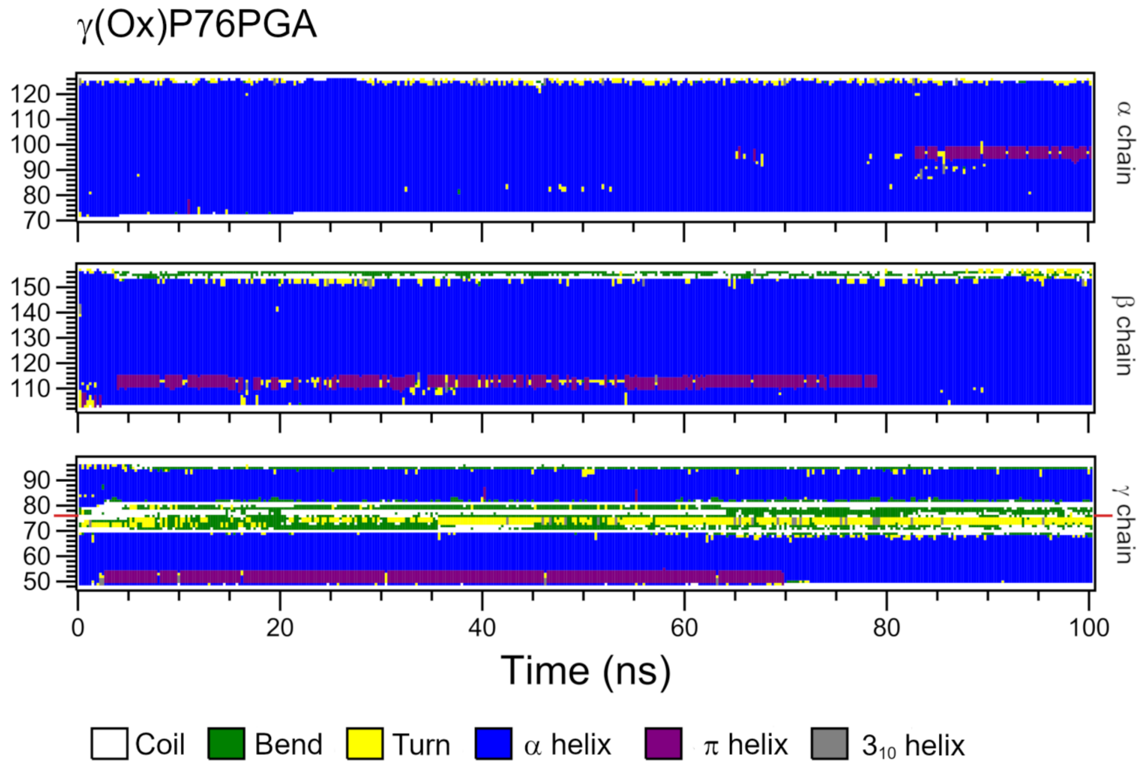

Fig S6. (all above) Development of secondary structure (DSSP) in time for coiled-coil connector systems. Positions of modified amino acids are highlighted by red bars at sides of plot.
