# Supplementary material for: Impact of posttranslational modifications on atomistic structure of fibrinogen
Source: PLoS One. 2020 Jan 29;15(1):e0227543. doi: 10.1371/journal.pone.0227543 (PMC6988951; doi:10.1371/journal.pone.0227543)
Supplement: S7 Fig — The decrease of the angle in Bβ(Ox)K122 and Aα(Ox)M91 may be caused by refolding of the α-helix. (PDF) [file pone.0227543.s009.pdf]

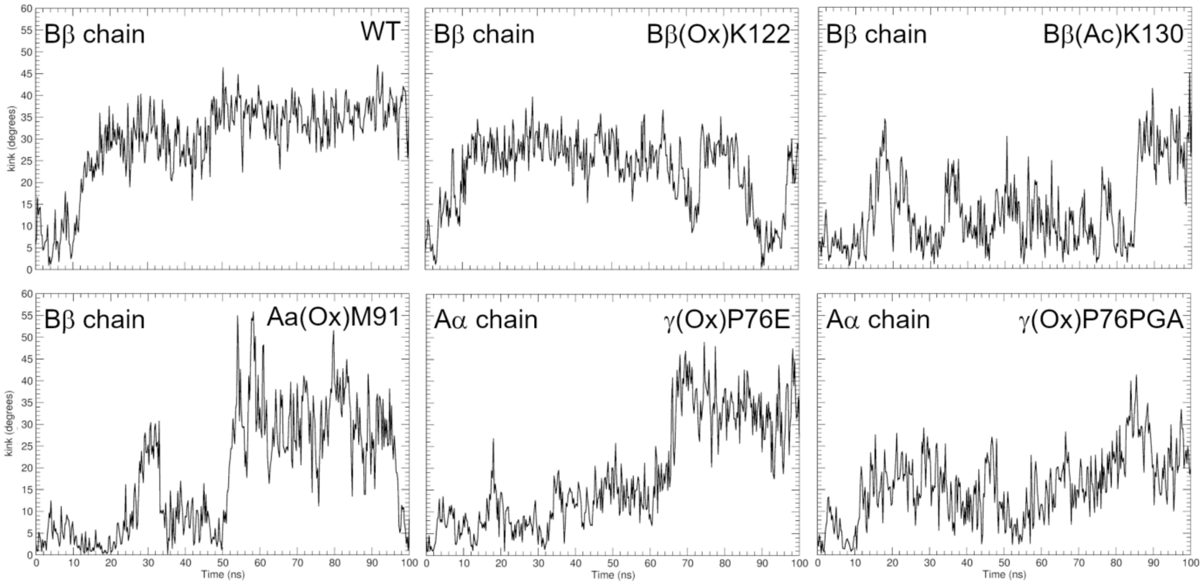

Fig S7. Characterization of kink induced into the α-helices by their partial switch to π-helices. The decrease of the angle in Bβ(Ox)K122 and Aα(Ox)M91 may be caused by refolding of the α-helix.
