# Supplementary material for: Impact of posttranslational modifications on atomistic structure of fibrinogen
Source: PLoS One. 2020 Jan 29;15(1):e0227543. doi: 10.1371/journal.pone.0227543 (PMC6988951; doi:10.1371/journal.pone.0227543)
Supplement: S10 Fig — Positions of the modified amino acids are highlighted by red bars at sides of plots. (PDF) [file pone.0227543.s012.pdf]

WT

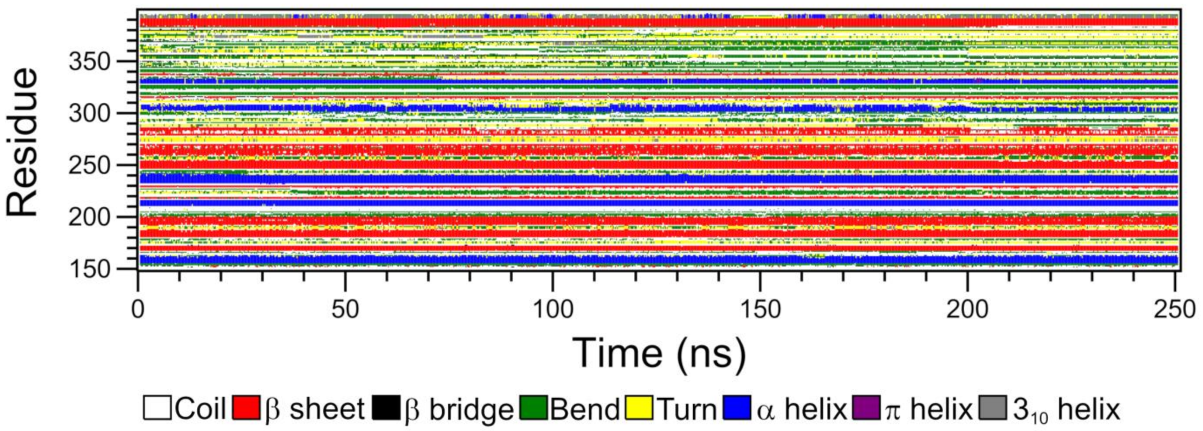

γ(Ox)R375

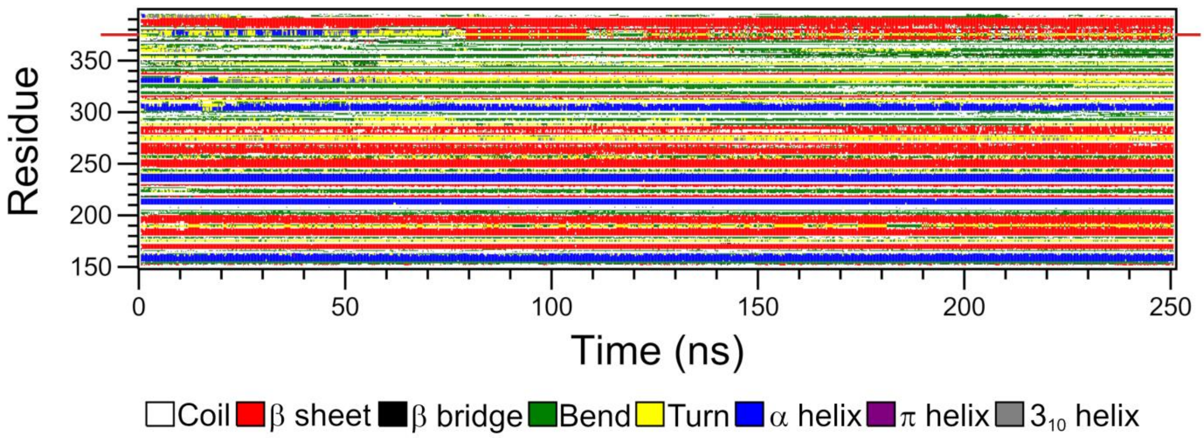

γ(Ox)K380

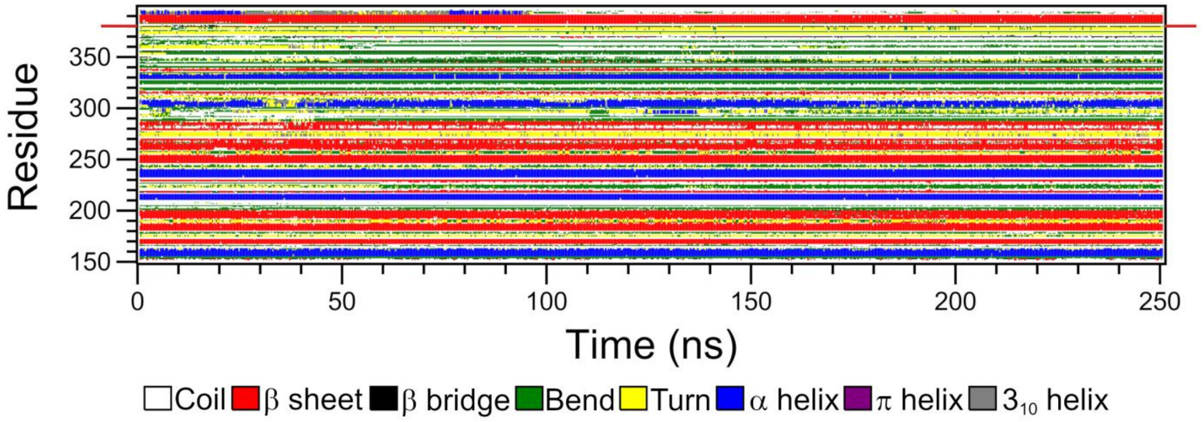

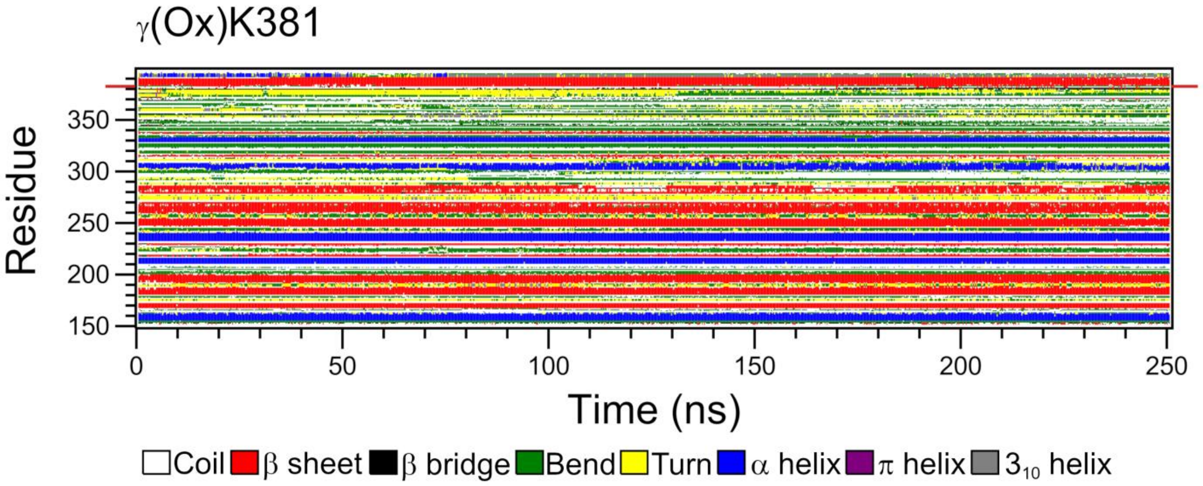

Fig S10. (all above) Development of secondary structure (DSSP) for systems examining γ-nodule of fibrinogen. Modified amino acid is highlighted by red bars at sides of plot.
