# Supplementary material for: Impact of posttranslational modifications on atomistic structure of fibrinogen
Source: PLoS One. 2020 Jan 29;15(1):e0227543. doi: 10.1371/journal.pone.0227543 (PMC6988951; doi:10.1371/journal.pone.0227543)
Supplement: S11 Fig — Distance of Ca2+ ion from Cα carbons of γD318 (black), resp. γD320 (red) and from C carbon of γF322 (green) in dependence of time. (PDF) [file pone.0227543.s013.pdf]

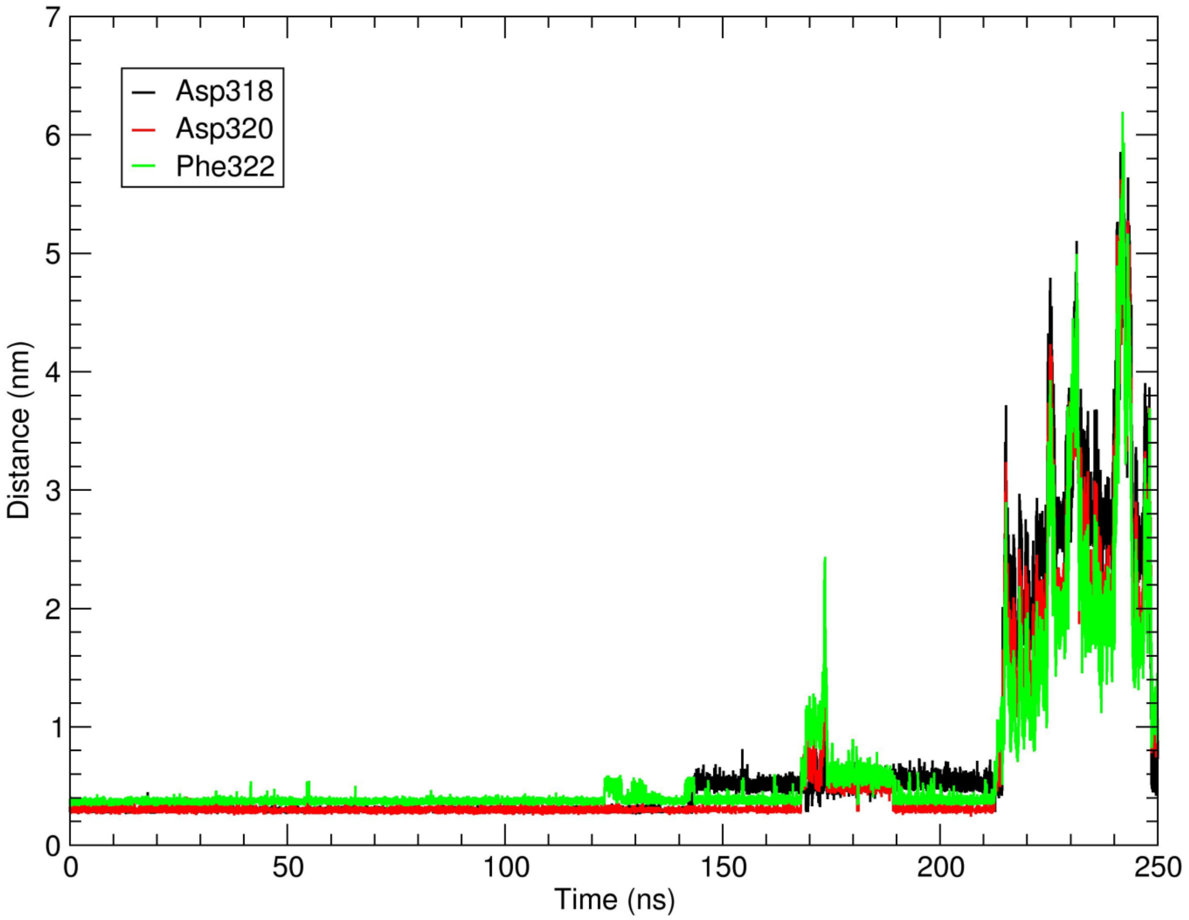

Fig S11. Unbinding of the Ca^{2+} ion. Distance of Ca^{2+} ion from C_α carbons of γD318 (black), resp. γD320 (red) and from C carbon of γF322 (green) in dependence of time.
