# Supplementary material for: Impact of posttranslational modifications on atomistic structure of fibrinogen
Source: PLoS One. 2020 Jan 29;15(1):e0227543. doi: 10.1371/journal.pone.0227543 (PMC6988951; doi:10.1371/journal.pone.0227543)
Supplement: S12 Fig — Green lines show hydrogen bonds formed in the C-terminal part of the system with oxidized γR375. In the WT simulation γR375 forms hydrogen bond only with γK373 (shown in violet). Carbon is shown in cyan, hydrogen in white, oxygen in red, nitrogen in blue and Ca2+ ion in black. (PDF) [file pone.0227543.s014.pdf]

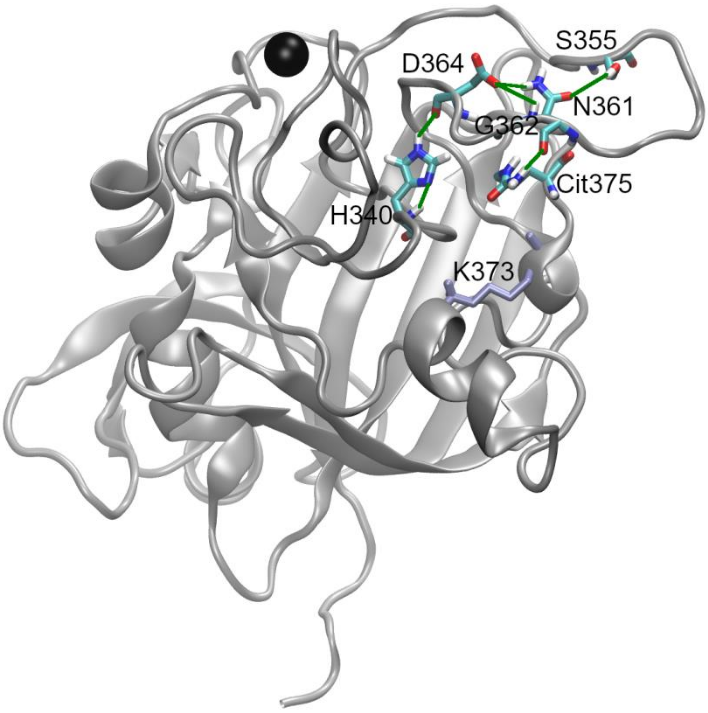

Fig S12. Hydrogen bond network formed as a result of oxidation of γR375. Green lines show hydrogen bonds formed in the C-terminal part of the system with oxidized γR375. In the WT simulation γR375 forms hydrogen bond only with γK373 (shown in violet). Carbon is shown in cyan, hydrogen in white, oxygen in red, nitrogen in blue and Ca^{2+} ion in black.
