# Supplementary material for: Impact of posttranslational modifications on atomistic structure of fibrinogen
Source: PLoS One. 2020 Jan 29;15(1):e0227543. doi: 10.1371/journal.pone.0227543 (PMC6988951; doi:10.1371/journal.pone.0227543)
Supplement: S13 Fig — (PDF) [file pone.0227543.s015.pdf]

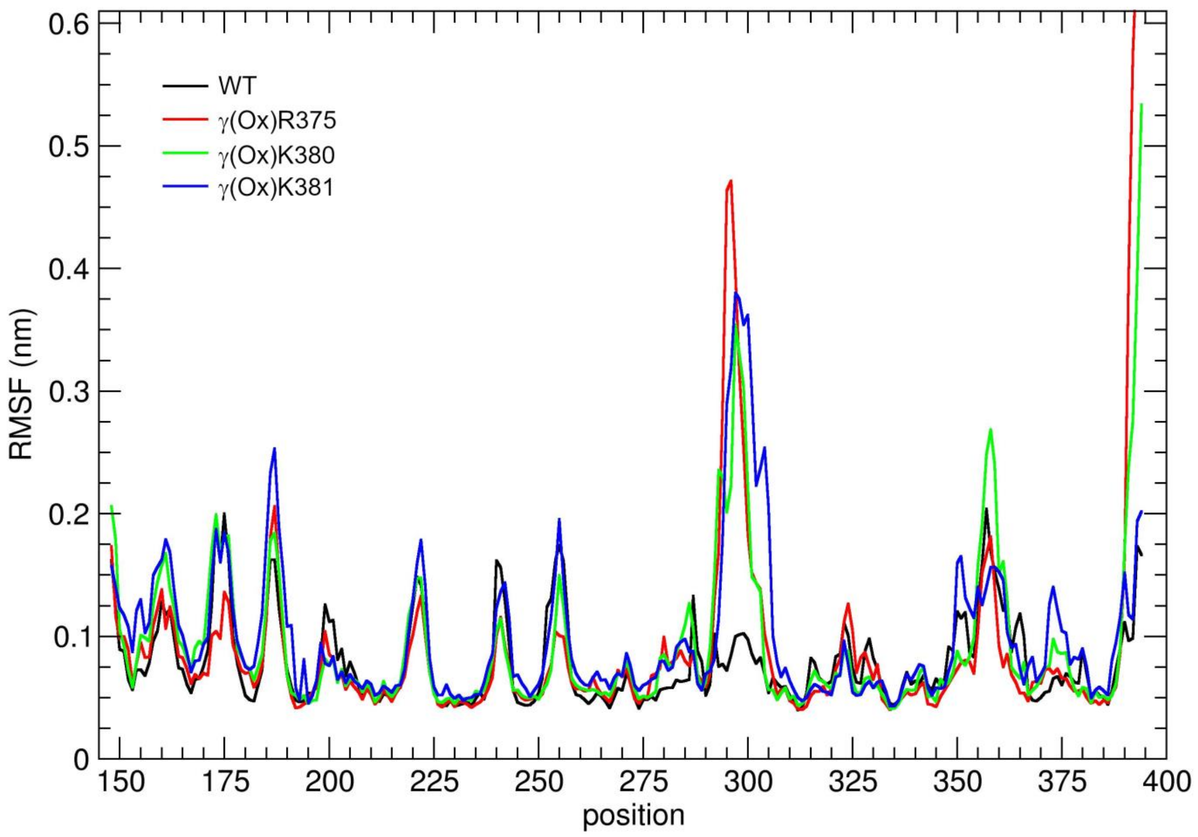

Fig S13. RMSF of C_α carbons computed over the last 25 ns of simulations of the γ-nodule systems.
