# Supplementary material for: Impact of posttranslational modifications on atomistic structure of fibrinogen
Source: PLoS One. 2020 Jan 29;15(1):e0227543. doi: 10.1371/journal.pone.0227543 (PMC6988951; doi:10.1371/journal.pone.0227543)
Supplement: S14 Fig — Ramachandran plot for amino acids γ50–γ90 of fibrinogen depicts γM78 in the region typical for collagen triple helix. The other amino acids nearby belong to the unfolded region of the γ chain. Plot was made by Procheck. (PDF) [file pone.0227543.s016.pdf]

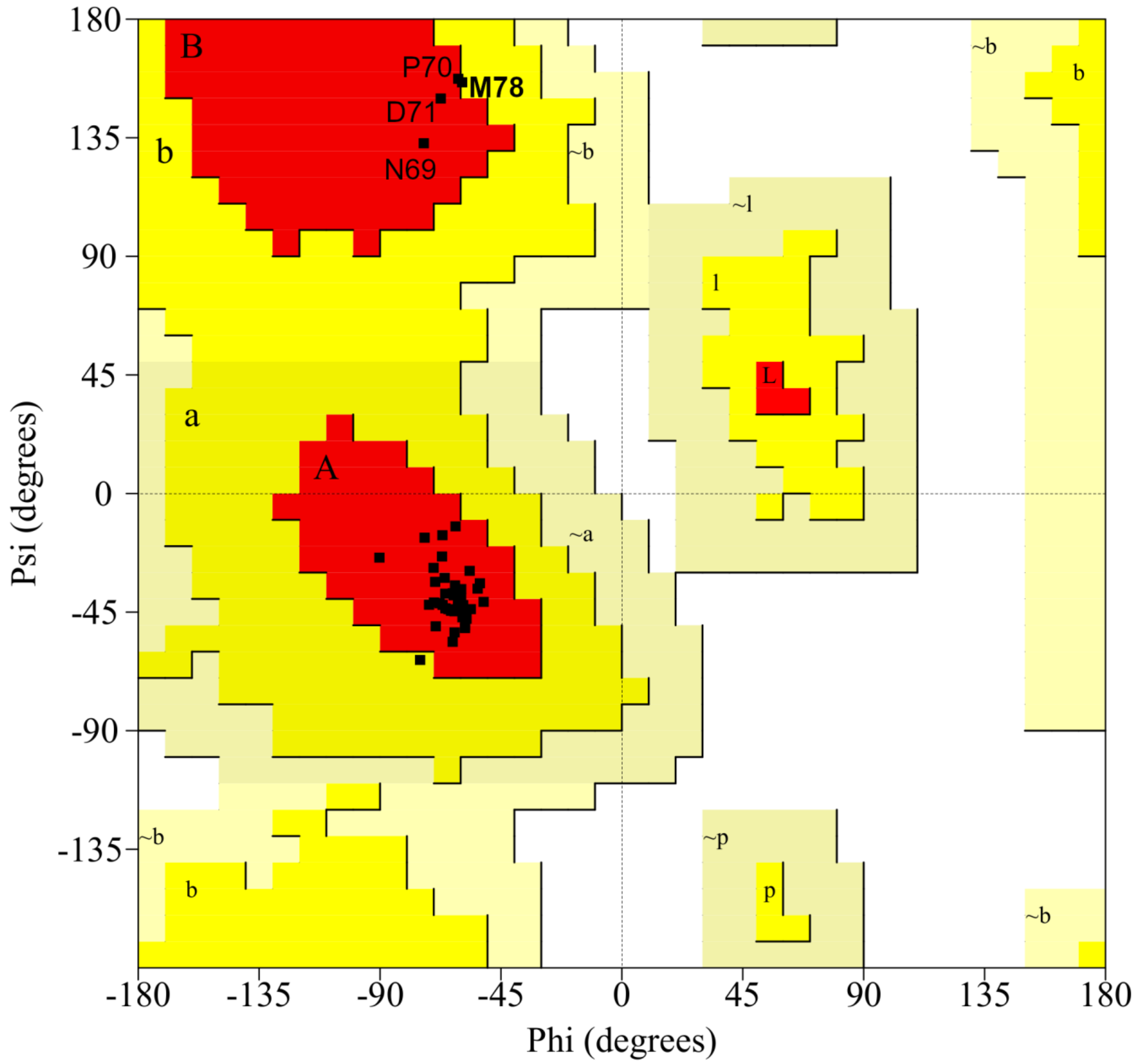

Fig S14. Ramachandran plot for selected amino acids of the γ chain. Ramachandran plot for amino acids γ50 to γ90 of fibrinogen shows γM78 in the region typical for collagen triple helix. The others amino acids nearby belongs to the unfolded region of the γ chain. Plot was made by Procheck [14].
